# Pathogenicity and Pre-Characterised Putative Effectors of *Fusarium oxysporum* and *F. proliferatum* in Garlic (*Allium sativum*) and Other *Allium* spp.

**DOI:** 10.3390/jof12040264

**Published:** 2026-04-06

**Authors:** Jessie Rose Harper, Saidi Achari, Tonga Li, Cherie Gambley, Stephen Harper, Victor Galea

**Affiliations:** 1 School of Agriculture and Food Sustainability, The University of Queensland, Gatton, QLD 4343, Australia; c.gambley@uq.edu.au (C.G.); s.harper1@uq.edu.au (S.H.); v.galea@uq.edu.au (V.G.); 2 Hawkesbury Institute for the Environment, Western Sydney University, Richmond, NSW 2753, Australia; 3 AgriBio, Agriculture Science and Technology, Bundoora, VIC 3083, Australia; saidi.achari@agriculture.vic.gov.au (S.A.); tongda.li@agriculture.vic.gov.au (T.L.)

**Keywords:** plant pathology, fungal plant pathogen, fusarium, allium, secreted in xylem, *SIX* genes

## Abstract

*Allium* spp. (alliums) are susceptible to rot-diseases caused by pathogenic *Fusarium* spp., including *F. proliferatum* (FP) and *F. oxysporum* (FO), which can cause severe crop losses. A series of pathogenicity tests of four FP isolates from garlic (*Allium sativum*), four FO isolates from garlic and three FO isolates from onion (*Allium cepa* var. *cepa*) were conducted on garlic seedlings and cloves, onion seedlings and bulbs, and shallot (*Allium cepa* var. *aggregatum*) bulbs to determine the virulence of the isolates. A combination of PCRs and whole-genome sequencing (WGS), using ONT long-read technology, was used to identify genes encoding putative effectors. The FP isolates caused moderate to severe symptoms in garlic and contained homologues of *SIX2*, *CRX1* and *CRX2*, and either *SIX9* or *SIX13.* The FOC *ex* onion isolates caused severe disease symptoms in all allium species tested, while FO from garlic caused moderate to severe disease in garlic but only mild symptoms in onion and shallot. *Fusarium oxysporum* f. sp. *cepae ex* onion potentially contained homologues of *SIX3*, *SIX5*, *SIX7*, *SIX9*, *SIX10*, *SIX12*, *SIX14*, *C5*, *CRX1* and *CRX2*. The most pathogenic FO isolate to garlic was Fo_VPRI44630 *ex* garlic, which contained *SIX9*, *SIX13*, *C5*, *CRX1* and *CRX2*. The difference in virulence and putative effector profiles suggests evidence of host-associated differentiation, and as such, the f. sp. or race designation between FO *ex* garlic and FO *ex* onion should be investigated further. This is an important finding for future research into best management practices and breeding for disease resistance to FO and FP in garlic.

## 1. Introduction

Garlic (*Allium sativum* L.) is an important crop grown worldwide for food and medicinal purposes [1]. Garlic is of the genus *Allium* (alliums), which also includes other important vegetables such as onion (*Allium cepa* var. *cepa* L.), shallot (*A. cepa* var. *aggregatum*), bunching onion (*Allium fistulosum* L.) and leek (*A. porrum* L.) [2]. Fusarium clove and bulb rot can cause severe crop losses in alliums, and many *Fusarium* species are attributed to causing this disease while *Fusarium proliferatum* (FP) [3,4,5,6] and *F. oxysporum* species complex (FO) [4,5,6,7] are the most dominant identified causal pathogens [8]. *Fusarium* spp. can cause disease during storage, particularly in garlic and onion, where symptoms of browning and rotting of bulb tissue occurs. *Fusarium* spp. also causes in-field disease symptoms in alliums including yellowing and twisting of leaves, which progresses to plant wilting. As the disease develops, it causes browning and rotting of the baseplate, bulbs, cloves, and roots and in severe cases can result in plant death [9].

*Fusarium proliferatum* is a significant pathogen worldwide with a wide host range including many important crop species, such as onion, maize (*Zea mays* L.), wheat (*Triticum aestivum* L.), and rice (*Oryza sativa* L.) [4,9]. *Fusarium proliferatum* is known to cause severe economic losses through postharvest diseases including Fusarium rot in garlic and in-field diseases such as vascular wilts, root rot, stem rot, and crown rot depending on the host species [9]. Importantly, FP is known to produce mycotoxins, including fumonisins, moniliformin, and beauvericin, that are toxic to humans and animals [4,9]. These mycotoxins can survive food processing procedures because they are chemically stable, which may be of significant concern to human health [4]. Host specificity has not currently been identified in FP, and FP *ex* garlic has been found to cause severe disease in multiple *Allium* spp. including onion and leek [9]. However, differences in disease severity have been observed depending on the species and cultivar [9].

While the *Fusarium oxysporum* species complex consists of pathogenic and non-pathogenic strains [10], a diverse range of non-pathogenic strains of FO are commonly found in the soil environment surviving as saprophytes and endophytes [11,12]. In contrast to FP, pathogenic FO cause disease in many crops, but strains are grouped together based on host specificity under the classification of *formae specialis* (f. sp.) [10]. Currently, there are arguably 106 *formae speciales* (ff. spp.) of FO, and 16 of these have been determined to have races [10]. The literature relating to ff. spp. of FO infecting *Allium* species, especially garlic, is inconsistent. Presently, *F. oxysporum* f. sp. *cepae* (FOC) is defined as the f. sp. causing disease in onion and other *Allium* species, but races have not been identified in this f. sp. [10,12,13,14,15]. *Fusarium oxysporum* f. sp. *garlic* has been identified as causing disease in garlic [5,7,10]; however, FO infecting garlic is often named FOC [16,17,18]. *Formae speciales* cannot be determined by standard molecular methods using multilocus housekeeping genes [12,19,20]. The pathogenicity of FP and FO is due to secreted effector proteins that allow fungi to infect and colonise plant tissue, subsequently causing disease [21]. Pathogenicity tests and the presence or absence of secreted putative effector genes, in particular the secreted in xylem (*SIX*) genes, and their DNA sequences have been used to discern some ff. spp. and races in FO [12,19,20].

Studies on FO f. sp. *lycopersici* in tomato (*Solanum lycopersicum*) plants identified the *SIX* genes and characterised some to be associated with pathogenicity of FO f. sp. *lycopersici* [22]. These *SIX* genes encode effector proteins that are associated with the pathogenicity of isolates; so far, 14 unique *SIX* genes have been identified [23]. Taylor et al. (2016) [12] further identified the novel putative effectors *C5*, *CRX1* and *CRX2* present in FOC. The *SIX* genes and important putative effectors recorded in FOC are *SIX3*, *SIX5*, *SIX7*, *SIX9*, *SIX10*, *SIX12*, *SIX14*, *C5*, *CRX1*, and *CRX2* [12,14,15,23], while for FP isolates the *SIX2* gene homologues *SIX2-1* and *SIX2-2* and *CRX2* [15,24] have been identified; however, the role of *SIX2* in the pathogenicity of FP in onion is not understood.

There is limited research on the cross-pathogenicity of FP and FO in garlic, onion and shallot [7]. This research addresses this gap in knowledge and examines the pathogenicity of FP and FO in *Allium* spp. Only limited research on the virulence-related genes of these pathogens has been conducted [12,14,15], and as such, this research aims to identify the genes associated with pathogenicity in FP and FO *ex* garlic and FO *ex* onion isolates.

## 2. Materials and Methods

### 2.1. Isolation of F. oxysporum and F. proliferatum

Garlic bulbs with symptoms of a dry rot and basal rot were identified and collected in Gatton, Queensland, Australia (27.54346° S, 152.32934° E), and affected bulbs had a pungent sulphur odour. Within each bulb the dry rot was not consistently visually present in every clove. Cloves that had a light brown lesion starting at the base plate and spreading up the clove were selected for pathogen isolation. A piece of the diseased clove, approximately 5 × 5 mm, that included the margin of the lesion was excised and surface sterilised by immersing the tissue in 70% ethanol for 3 min. The tissue was then placed onto a Petri dish with 1/4 strength PDA and 100 µg mL^−1^ streptomycin and incubated in the dark at 25 °C for 5–7 days, then stored at 4 °C. The single spore isolation technique as described by Choi, Hyde [25] was used to make pure cultures of the isolates. Briefly, a small piece of the agar plate culture with the isolate was placed into sterile distilled water and vigorously shaken to mix the solution. The extract was adjusted to a concentration of 400 conidia per mL of water, and a 500 µL aliquot was pipetted onto a water agar plate. After 12 to 24 h incubation the spores germinated, and a single germinated spore was selected under a dissecting microscope and subcultured using a sterile needle. A total of ten germinated spores were selected and distributed across two plates. After three days of growth, when colonies were 1–2 cm, a further subculture was made to give a pure culture of the isolate. This process was repeated for each isolate, and pure cultures were used for pathogenicity tests and subsequent experiments. The initial identification of the isolates as *Fusarium* was made based on morphology (Supplementary Table S1).

Additionally, isolates of FOC *ex* onion were obtained from the South Australian Research and Development Institute (SARDI) (Fo_VPRI44637 *ex* onion and Fo_VPRI44638 *ex* onion) and the Enza Zaden Australia (Narromine, NSW, Australia) seed company (Fo_VPRI44636 *ex* onion).

### 2.2. DNA Extraction and Sequencing for Species Identification

A DNeasy Plant Pro kit (Qiagen, Hilden, Germany) was used according to the manufacturer’s instructions to extract DNA from the *Fusarium* isolates. A portion of the translation elongation factor 1-alpha (TEF) was used to identify the species of *Fusarium* isolates by PCR amplification and subsequent amplicon sequencing using the primers EF1 (5′-ATGGGTAAGGARGACAAGAC-3′) and EF2 (5′-GGARGTACCAGTSATCATG-3′) [26]. The 25 µM PCR reaction contained 12.5 µM 2× Concentration Promega PCR Master mix (final concentration of 1×), both primers at 0.5 µM, 5 µL DNA template, and nuclease-free water to make up the volume. The PCR reactions were 1 cycle of 95 °C for 1 min; 30 cycles of 95 °C for 10 s, 52 °C for 10 s and 72 °C for 30 s, followed by 1 cycle of 72 °C for 2 min. PCR amplicons were visualised by gel electrophoresis, then purified by ExoSAP-IT™ Express PCR Product Cleanup (Thermo Fisher Scientific, Waltham, MA, USA).

Purified PCR products were then sequenced in both directions using primers EF1, EF2, EF-3 (5′-GTAAGGAGGASAAGACTCACC-3′) and EF-22T (5′-AGGAACCCTTACCGAGCTC-3′) [27] at the Genetic Research Services (The University of Queensland), Queensland, Australia. Consensus sequences were generated using Geneious Prime (version 2023.1.2) and identified using BLASTn search [28] of the NCBI database (https://blast.ncbi.nlm.nih.gov/Blast.cgi?PROGRAM=blastn&PAGE_TYPE=BlastSearch&LINK_LOC=blasthome, accessed 30 July 2023) [29] for the highest identity. The TEF sequences were then submitted to the GenBank (Supplementary Table S1).

### 2.3. Pathogenicity Assay Methods

Seven experiments were used to assess the cross-pathogenicity of FP *ex* garlic, FO *ex* garlic and FOC *ex* onion on garlic, onion and shallot, and the details for each are displayed in Table 1. Experiments 1 and 2 assessed the pathogenicity of *Fusarium* using the isolates derived from the affected garlic cloves and were conducted on both garlic cloves in storage and garlic seedlings. The four FP isolates were Fp_VPRI44629, Fp_VPRI44631, Fp_VPRI44632 and Fp_VPRI44633, and four FO isolates were Fo_VPRI44628, Fo_VPRI44630, Fo_VPRI44634, and Fo_VPRI44635. The pathogenicity assays for in-storage assay on bulbs/cloves and on seeds/seedlings test different pathogenicity mechanisms [30]. Thus, both types of pathogenicity assays were used in this study. Experiment 1 examined the pathogenicity of FO and FP isolates on garlic cloves in storage. Experiment 2 examined the pathogenicity of FO and FP isolates on garlic seedlings using disease ratings.

Experiments 3–7 examined the cross-pathogenicity of FO *ex* garlic and FOC *ex* onion on garlic, onion and shallot. Four FO from garlic (Fo_VPRI44628, Fo_VPRI44630, Fo_VPRI44634, and Fo_VPRI44635) and three FOC isolates from onion (Fo_VPRI44636, Fo_VPRI44637, and Fo_VPRI44638) were used in these pathogenicity assays. Experiment 3A and 3B assessed the pathogenicity of FO isolates on garlic cloves in storage. Experiment 4 assessed the pathogenicity of FO isolates on garlic seedlings. Experiment 5 assessed the pathogenicity of FO isolates on shallot bulbs in storage. Experiment 6 evaluated the pathogenicity of FO isolates on onion bulbs in storage. Finally, Experiment 7 examined the pathogenicity of FO isolates on onion seedlings.

#### 2.3.1. General Experimental Method for Pathogenicity Assays

##### Surface Sterilisation of Plant Material

Surface sterilisation of garlic cloves, shallot bulbs, and onion bulbs and seeds were conducted prior to use in pathogenicity experiments. The garlic cloves, shallot bulbs and onion bulbs were washed in deionised water for 20 min, surface sterilised with 1% NaOCl for three minutes, and washed five times in sterile water, then allowed to dry overnight. The onion seeds were surface sterilised with 1% NaClO for one minute and washed five times in sterile water, then allowed to dry overnight.

##### Inoculum Preparation

Multiple pathogenicity assays were used in this study, which were modified from methods described by Haapalainen et al. [14,15,30] for onion and garlic. Conidial spore suspensions for all experiments were prepared as pure cultures of the isolates grown for 18 days on ¼ strength PDA plates; the cultures were then scraped with a sterile scalpel, suspended in 30 mL of sterile distilled water, agitated, and filtered through four layers of sterilised gauze. The conidial density was determined by a Neubauer Haemocytometer Cell Counting Chamber and adjusted to 10^6^ conidia per mL.

##### Disease Rating Assessment

For Experiment 1, 3A and 3B, which were conducted on garlic cloves in storage, the disease ratings (DR) were assigned to individual cloves as follows: DR 0—No disease symptoms. DR 1—Mild symptoms included discolouration at the site of injection (up to 10% of the clove displaying discolouration). DR 2—Moderate symptoms consisted of discolouration beyond the site of injection (~10–30% of clove having rot symptoms). DR 3—Moderate to severe symptoms consisted of cloves with 30–50% of the clove having discolouration/rotting symptoms and severe symptoms. DR 4—Highly severe disease symptoms consisted of 50–100% rotting of the clove.

For Experiment 2 and 4, which were conducted on garlic seedlings, the disease ratings were assigned to individual seedlings as follows: DR 0—No disease symptoms. DR 1—Mild symptoms included up to 10% of the clove having discolouration and usually at the baseplate. DR 2—Moderate symptoms consisted of ~10–30% of the clove having rot symptoms. DR 3—Moderate to severe symptoms consisted of cloves with 30–50% of the clove having discolouration/rotting symptoms. DR 4—Highly severe disease symptoms consisted of 50–100% rotting of the clove.

For Experiment 5, which was conducted on mature shallot bulbs, and Experiment 6, which was conducted on mature onion bulbs, the disease ratings were assigned to individual bulbs as follows: DR 0—No disease symptoms. DR 1—Mild symptoms included discolouration at the site of injection (up to 10% of the bulb displaying discolouration). DR 2—Moderate symptoms consisted of discolouration beyond the site of injection (~10–30% of bulb having rot symptoms). DR 3—Moderate to severe symptoms consisted of bulbs with 30–50% of the clove having discolouration/rotting symptoms. DR 4—Highly severe disease symptoms consisted of 50–100% rotting of the bulb.

##### Statistical Analysis

The statistical analyses for all experiments were completed using R version 4.3.3 [31]. The results for disease ratings in Experiment 1, 2, 3A, 3B, 4, 5 and 6 were analysed using ordinal logistic regression with the MASS package [32] in R. The model was polr (formula = Disease rating ~ Treatment, Hess = TRUE), and the isolates were compared by their 95% confidence intervals of the log odds ratio. Conversely, the results of cumulative germination in Experiment 7 were analysed using logistic regression (family = quasibinomial) in R. The results of logistic regression on seed germination for FO isolates were compared using the package emmeans [33] in R and grouped using the multcomp package [34] in R.

### 2.4. DNA Extraction, Library Preparation and Sequencing

Genomic DNA was extracted from pure fungal cultures grown on 1/4 PDA for two weeks and stored at 4 °C for two weeks. A ZymoBIOMICS DNA Miniprep Kit (Zymo Research, Irvine, CA, USA) was used to extract DNA as per the manufacturer’s instructions, and the quantity was assessed using a Quantus™ Fluorometer (Promega, Madison, WI, USA). DNA was stored at −20 °C until library preparation and sequencing.

Genomic DNA was sequenced using long read sequencing technology. A library was prepared using an Oxford Nanopore Technologies (ONT) Native Barcoding kit 24 V14 (SQK-NBD114.24, ONT, Oxford, UK) and sequenced on a MinION Mk1C platform with R10.4.1 Flow Cell (FLO-MIN1.14). Dorado (version 0.5.3) with the super high accuracy model was used for basecalling and demultiplexing of ONT genomic sequence data (raw read signals), and for trimming of sequencing adapters and barcodes. The cleaned reads were de novo assembled using Flye assembler (version 2.9.3) with three iterations and a read error of 0.03 [35].

### 2.5. RNA Extraction, Library Preparation and Sequencing

RNA was extracted from pure fungal cultures grown on ¼ PDA for six days using ZymoBIOMICS RNA Miniprep Kit (Zymo Research, USA), which was used according to the label instructions. The quality and quantity of RNA was assessed by NanoDrop Spectrophotometer (Thermo Fisher Scientific, USA) and visualised on Agilent 4200 TapeStation (Agilent Technologies, Santa Clara, CA, USA). RNA clean-up was completed using ProN*ex* Size-Selective Purification System (Promega, USA). The RNA was stored at −80 °C and transported on dry to AGRF in Melbourne, Australia, for sequencing using NovaSeq 150 bp PE sequencing on the Illumina NovaSeq X platform (Illumina, San Diego, CA, USA) with 50 M depth per sample. For isolates Fp_VPRI44629, Fp_VPRI44631, Fp_VPRI44632, Fp_VPRI44633 and Fo_VPRI44630, stranded RNAseq libraries were prepared using mRNA enriched via polyA selection, and for isolates Fo_VPRI44634, Fo_VPRI44635, and Fo_VPRI44628, stranded RNAseq libraries were prepared using mRNA enriched via rRNA depletion. Sequencing adapters, barcodes and low-quality reads were trimmed from RNA Illumina sequence data using fastp (version 0.21.0) [36]. De novo assembly of expressed transcripts were performed using SPAdes assembler (version 3.15.5) [37]. HISAT2 (version 2.2.1) was used to complete the alignment of clean RNAseq reads to the assembled genomes [38].

### 2.6. Genome Assembly, Annotation and Classification

Funannotate (version 1.8.17) was used for gene prediction and annotation of the genomes based on DNA and RNA data [39]. The funannotate pipeline called the resources merops (12.0), uniprot (2023_03), repeats (1.0), and mibig using diamond (version 2.1.8); dbCAN (12.0), and pfam (35.0), using hmmer3 (version 3.3.2); and interpro (version 5.66) Busco (version 2.0), and gene2product (version 1.91). The genomes were submitted to GenBank, and accessions numbers were created.

### 2.7. Identification of Known Putative Effector Genes and Housekeeping Genes in Genomes

Specific putative effectors and housekeeping genes were identified by using BLASTn (version 2.15.0) search of a reference sequence, and the corresponding sequence was extracted from the genomes. The reference sequences used in this study are present in Supplementary Table S2.

### 2.8. PCR-Based Molecular Identification of Known Putative Effector Genes

To confirm the absence of the putative effectors in the genomes of FP, FO *ex* garlic, and FO *ex* onion isolates, a PCR-based molecular method was used. Primers published by Taylor et al. [12] and Haapalainen et al. [15] that were previously used to identify *SIX* genes, *CRX1*, *CRX2* and *C5*, in FOC isolates (Table 2) were used. The PCR reaction consisted of 12.5 µL Promega PCR mastermix, 0.05 µM forward primer, 0.05 µM reverse primer, and 50–250 ng DNA template, made up to 25 µL reaction volume with nuclease-free water. The PCR thermocycling conditions were 1 cycle at 94 °C for 2 min, 30 cycles of 45 s at 94 °C, 30 s annealing temperature, 1 min at 72 °C, followed by 1 cycle of 72 °C for 5 min.

### 2.9. Sequencing of SIX9 from F. oxysporum f. sp. cepae Isolates

To determine the sequence of *SIX9* in the FOC isolates from onion Fo_VPRI44636, Fo_VPRI44637, and Fo_VPRI44638, the clean PCR products were sequenced in both directions using primers FOCSIX9F (5′-GGCCCAGCCCTAGTCTAACTCC-3′) and FOCSIX9R (5′-AACTTAACATGCTGGCCGTCAATCG-3′) at the Genetic Research Services (The University of Queensland), Queensland, Australia. Consensus sequences were created using Geneious Prime (version 2025.0.3).

### 2.10. Phylogenetic Analysis and Tree Building

The translation elongation factor (*TEF*) 1α, DNA-directed RNA polymerase II largest subunit (*RPB1*), DNA-directed RNA polymerase II second largest subunit (*RPB2*) and beta-tubulin (*TUB*) genes were selected as phylogenetic loci to infer the phylogenetic relationships of the isolates. FP and FO genomes were downloaded from GenBank and used to compare to the genomes of FO and FP isolated from garlic in this study, and the accession numbers are presented in Supplementary Table S2. Reference sequences (Supplementary Table S2) were used to identify each locus from the FO and FP isolates included in the phylogenetic tree. A BLASTn (version 2.15.0) search was completed for each reference locus sequence in all FO and FP genomes. The identified sequences were concatenated in Geneious Prime (version 2025.0.3) and aligned using Clustal Omega(version 1.2) within Geneious Prime, and gaps were removed, which resulted in a sequence alignment of 4251 base pairs (bp) (*TEF*, 567 bp; *RPB1*, 1604 bp; *RPB2*, 845 bp; and TUB, *1148* bp). *Fusarium avenaceum* (isolate WV21P1A; GenBank accession: CP109663.1-CP109671.1) was used as an outgroup.

The phylogenetic analysis of the concatenated *TEF*/*RPB1*/*RPB2*/*TUB* data set was completed using IQ-TREE (version 2.3.6), which estimates maximum likelihood (ML) phylogenies [41]. The concatenated alignment was partitioned [42] into the 4 loci, and the in-built function ModelFinder [43] within IQ-TREE was used to determine the best models. Branch support was assessed using 1000 bootstraps [44]. Branch support was also assessed using SH-like approximate likelihood ratio test (Guindon et al., 2010 [45]) (SH-aLRT) within IQ-TREE with 1000 bootstrap replicates. The resulting consensus tree was viewed and edited in FigTree (version 1.4.4).

Phylogenetic trees were also constructed for specific putative effectors, including *SIX2*, *SIX9*, *SIX13*, *CRX1*, *CRX2* and *C5*, which were identified in the FP and FO genomes in this study. Nucleotide alignments were generated from the consensus sequences of *SIX9* from the FOC isolates, and the sequence was retrieved after BLASTn (version 2.15.0) results for the putative effectors (Supplementary Table S3) of whole-genome analysis of the isolates. *CRX1*, *CRX2*, *C5* and *SIX* gene sequence data was downloaded from GenBank, and the accession numbers are in Supplementary Table S4. There were 79 *Fusarium* spp. genomes downloaded from the GenBank, and a BLASTn (version 2.15.0) search of the putative effectors was conducted. The sequences of these putative effectors identified were added to gene trees for comparison. Nucleotide sequence alignments were conducted using Clustal Omega within Geneious Prime and were manually inspected, and gaps were removed from the ends. Gene trees were generated using IQ-TREE (version 2.3.6) [41], and ModelFinder [43] within IQ-TREE was used to determine the best model for each tree. Branch support was assessed using 1000 bootstraps [44] and SH-aLRT [45] with 1000 bootstrap replicates within IQ-TREE. The resulting consensus tree was viewed and edited in FigTree.

## 3. Results

### 3.1. Genome Sequence, Assembly, Statistics and Annotation

**Figure 1:**
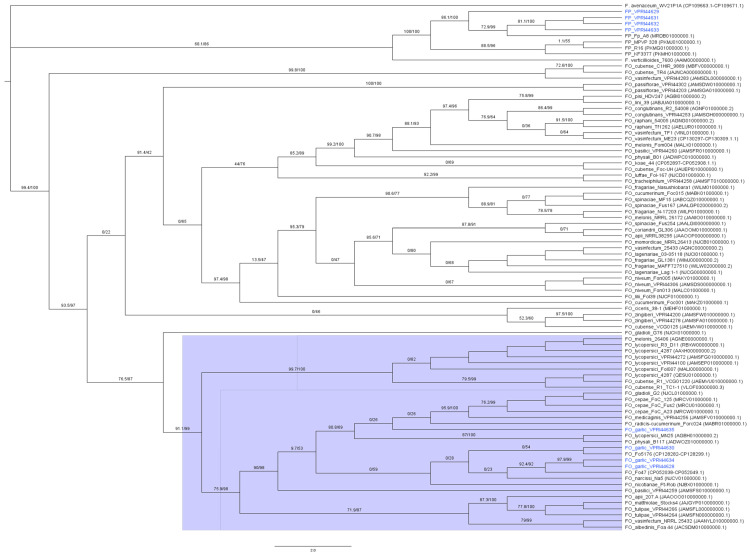
Maximum likelihood phylogenetic tree (transformed cladogram) of the concatenated gap-free alignment of TEF, RPB1, RPB2, and TUB loci for the four FO isolates from garlic and four FP isolates from garlic in this study, as well as publicly available genomes of *F. avenaceum*, *F. verticillioides*, FP and FO isolates that are described in Supplementary Table S3. The isolates in blue were sequenced in this study.

The isolates obtained from diseased garlic were identified as *Fusarium* spp. based on their morphology (Supplementary Table S1) and molecular TEF sequences prior to WGS. *Fusarium oxysporum* isolates from garlic Fo_VPRI44630, Fo_VPRI44634, Fo_VPRI44635, and Fo_VPRI44628 were assembled into 210, 153, 24 and 160 contigs, respectively. RNA sequencing improved annotation of genes as shown in Table 3. The *Fusarium proliferatum* isolates from garlic Fp_VPRI44629, Fp_VPRI44631, Fp_VPRI44632 and Fp_VPRI44633 were assembled into 149, 211, 211 and 153 contigs (Figure 1).

### 3.2. Pathogenicity Experiments

The images in Supplementary Figures S1–S6 indicate the rating scale for disease severity.

#### 3.2.1. Effects of *Fusarium oxysporum* and *F. proliferatum ex* Garlic on Garlic (Experiments 1 and 2)

The results from the garlic clove pathogenicity testing (in storage) show that all isolates were significantly different from the control treatment (*p* < 0.05) (Table 4). All isolates in this study were successfully reisolated from the inoculated cloves, therefore fulfilling Koch’s postulates.

The results of Experiment 1 indicated isolate Fo_VPRI44630 *ex* garlic was the most pathogenic isolate to garlic and significantly more pathogenic than the other three FO isolates (95% confidence interval). Isolates Fp_VPRI44631, Fp_VPRI44629 and Fp_VPRI44633 had intermediate pathogenicity, whereas Fo_VPRI44635 was the least pathogenic and consistently had the lowest disease rating across all garlic pathogenicity experiments. Interestingly, Fp_VPRI44629 *ex* garlic was isolated from a healthy clove with no visible lesions and was the 2nd most pathogenic isolate in the clove storage assay. In Experiment 1, the FP isolates had less variation in disease severities (2.1–2.6) on garlic compared to the FO (0.9–3.5) (Table 4).

In Experiment 2 (garlic seedling inoculation), all isolates gave significantly higher disease ratings than the control treatment. However, there were no significant differences in disease ratings between isolates reflecting a higher variability than that recorded for the in-storage experiment (Table 4). However, the highest disease rating score was recorded for isolate Fo_VPRI44630 *ex* garlic, which was consistent with the results for the in-storage experiment.

#### 3.2.2. Effects of *Fusarium oxysporum ex* garlic and *F. oxysporum ex* Onion on *Allium* spp. (Experiments 3–7)

In experiments 3, 4, 5, and 6, Koch’s postulates were successfully fulfilled for the four isolates of FO *ex* garlic and the three isolates of FOC *ex* onion. The results demonstrate cross-pathogenicity of FO *ex* garlic and *ex* onion on both garlic and onion plants. The ratings of disease symptoms caused by FO *ex* garlic and FOC *ex* onion on garlic cloves in storage are presented in Table 5.

All FO *ex* garlic isolates and FOC *ex* onion isolates gave higher disease severity than the control treatment in garlic cloves in storage, and the results for each isolate were closely similar for varieties Glenlarge and Argentina and consistent with the results of Experiment 1. Fo_VPRI44630 *ex* garlic was the most pathogenic isolate, and in Experiment 3A was found to cause significantly greater disease symptoms than Fo_VPRI44628 *ex* garlic, and Fo_VPRI44635 *ex* garlic. Isolate Fo_VPRI44635 *ex* garlic was the least pathogenic isolate, which was also demonstrated in Experiment 1, and caused significantly less severe disease symptoms than Fo_VPRI44638 *ex* onion and Fo_VPRI44630 *ex* garlic (Table 5). In Experiment 3B, Fo_VPRI44630 *ex* garlic was significantly more pathogenic than Fo_VPRI44634 *ex* garlic, Fo_VPRI44635 *ex* garlic, and Fo_VPRI44637 *ex* onion. Isolate Fo_VPRI44635 *ex* garlic was the least pathogenic isolate in experiment 4B and had a significantly lower disease rating than Fo_VPRI44630 *ex* garlic and Fo_VPRI44638 *ex* onion (Table 5). The FOC *ex* onion plants (Fo_VPRI44636, Fo_VPRI44638 and Fo_VPRI44637) were highly pathogenic to garlic in the clove storage assay in both experiments 3A and 3B (Table 5).

In Experiment 4, which examined the pathogenicity of FO on garlic seedlings, all isolates with the exception of Fo_VPRI44635 had significantly greater disease symptoms than the uninoculated control (95% confidence interval). There was no significant difference between isolates in this experiment, but the highest disease severity rating value was recorded for isolate Fo_VPRI44630 *ex* garlic, while isolate Fo_VPRI44635 *ex* garlic had the lowest disease rating value and was not different from that in the control treatment (Table 5).

In Experiment 5, the most pathogenic isolates in mature shallot bulbs were Fo_VPRI44636 *ex* onion and Fo_VPRI44638 *ex* onion (Table 5), which gave higher disease severity ratings than all other FO isolates. The disease rating for Fo_VPRI44637 *ex* onion gave an intermediate disease rating that was higher than that of Fo_VPRI44628 *ex* garlic, Fo_VPRI44634 *ex* garlic, Fo_VPRI44630 *ex* garlic and the control treatment. The disease ratings of isolates Fo_VPRI44634 *ex* garlic and Fo_VPRI44628 *ex* garlic were not significantly different from the control treatment. The FOC *ex* onion isolates had significantly higher pathogenicity than the FO *ex* garlic in shallot bulbs. The FO *ex* garlic had no to very low pathogenicity in mature shallot bulbs.

In Experiment 6, onion bulbs of the highly susceptible variety ONAUS21.17630 were inoculated with FO, and the isolates had varying results of pathogenicity while control plants did not have rotting symptoms in onion bulbs (Table 5). The pathogenicity results of isolates on mature onion and shallot bulbs differed, which was evident as the disease ratings of Fo_VPRI44634 *ex* garlic and Fo_VPRI44628 *ex* garlic were not significantly different from control bulbs in shallots but caused mild symptoms in onion bulbs. In Experiment 6, Fo_VPRI44636 *ex* onion and Fo_VPRI44638 *ex* onion were significantly more pathogenic than Fo_VPRI44634 *ex* garlic, and Fo_VPRI44628 *ex* garlic. In both onion and shallot bulbs, Fo_VPRI44636 *ex* onion and Fo_VPRI44638 *ex* onion had the most severe disease symptoms and had significantly higher disease ratings than Fo_VPRI44634 *ex* garlic, Fo_VPRI44628 *ex* garlic and control treatments.

In Experiment 7, onion seeds that were inoculated with FO had significantly reduced germination compared to control plants (Table 6). All FO isolates, originating from both garlic and onion, were highly pathogenic to onion seed/seedlings. Isolate Fo_VPRI44637 *ex* onion significantly reduced germination compared to Fo_VPRI44635 *ex* garlic and control plants. Control plants had germination of 53% of seeds, and the lowest germination was in Fo_VPRI44637 *ex* onion with 4% of seeds germinating. Isolate Fo_VPRI44635 *ex* garlic had the highest germination of the FO isolates with 18%, although germination was not significantly different from isolates Fo_VPRI44630 *ex* garlic, Fo_VPRI44634 *ex* garlic, Fo_VPRI44628 *ex* garlic, Fo_VPRI44636 *ex* onion, or Fo_VPRI44638 *ex* onion. The results of Experiment 3, 4, 5, 6, and 7 show that both FO *ex* garlic and FOC *ex* onion were able to cause disease in garlic, onion and shallot plants. Although FOC isolates *ex* onion were highly pathogenic on garlic, onion and shallots, the level of pathogenicity of FO *ex* garlic varied in the different *Allium* species garlic, onion and shallots. Isolate Fo_VPRI44630 *ex* garlic, which caused severe disease symptoms and had the highest disease rating in garlic seedlings and cloves in storage, caused minor rotting in shallot bulbs, mild–moderate symptoms in onion bulbs and reduced germination of onion seeds to 8% compared to 53% germination in controls. Isolates Fo_VPRI44634 *ex* garlic and Fo_VPRI44628 *ex* garlic caused moderate disease symptoms in garlic cloves, mild symptoms in mature onion bulbs, no symptoms in mature shallot bulbs and reduced the germination of onion seed to 4% and 5%, respectively, compared to 53% in controls. Isolate Fo_VPRI44635 *ex* garlic had mild–moderate symptoms in pathogenicity tests in garlic, shallot and onion plants and significantly reduced the germination of onion seeds compared to control plants.

### 3.3. Pre-Characterised Putative Effectors

There have been several putative effectors identified in the literature as important for pathogenicity in *Fusarium* species. In this study, whole-genome sequencing was used to identify the pre-characterised putative effectors using BLAST+ (version 2.15.0) searches for the FP and FO *ex* garlic isolates used in pathogenicity tests. PCRs of the target genes encoding putative effectors were then used to confirm the presence and absence of the genes identified by WGS. Genomes of FO *ex* onion were downloaded from Genbank to compare the putative effector profiles, also using a BLAST search of the genomes. For the FO f. sp. *cepae ex* onion isolates used in the pathogenicity tests, only PCRs of the target genes were used to confirm the presence and absence of the putative effectors in FO *ex* garlic and FO ex onion. Thus, the FO *ex* garlic isolates were more deeply characterised than FO *ex* onion in this study. Of the FO isolates from garlic, Fo_VPRI44630 *ex* garlic was the most pathogenic and contained the most pre-characterised putative effectors compared to the other three isolates. The identified putative effectors included *SIX9*, *SIX13*, *C5*, *CRX1* and *CRX2* (Table 7). *Fusarium oxysporum* isolates Fo_VPRI44634 *ex* garlic and Fo_VPRI44628 *ex* garlic contained *SIX9*, *SIX13*, *CRX1* and *CRX2*. These isolates were intermediate in pathogenicity to Fo_VPRI44630 *ex* garlic and the least pathogenic isolate, Fo_VPRI44635 *ex* garlic, and only had one pre-characterised putative effector; *CRX1* identified by WGS and PCR analysis. Three genomes of FOC were downloaded from GenBank, and the pre-characterised putative effector profile was compared to the FO isolates from garlic in this study. The putative effector profile was different for the onion FO isolates compared with the garlic isolates. *SIX13* was specific to the isolates from garlic. In contrast, the onion isolates contained *SIX3*, *SIX5*, *SIX7*, *SIX10*, *SIX12*, and *SIX14* which were not present in the garlic isolates. The garlic and onion isolates shared the putative effectors *SIX9*, *C5*, *CRX1* and *CRX2*. Following this result, Australian FOC isolates from onion hosts were sourced, pathogenicity tests were conducted, and PCRs were used to identify the putative effectors present. In FOC isolated from onion Fo_VPRI44636 *ex* onion, Fo_VPRI44637 *ex* onion and Fo_VPRI44638 *ex* onion, the putative effectors *SIX5*, *SIX7*, *SIX9*, *SIX10*, *SIX12*, and *SIX14* were present. In addition to those *SIX* genes, Fo_VPRI44637 *ex* onion and Fo_VPRI44636 *ex* onion also harboured *SIX3* and *C5*. In addition to the *SIX* genes and *C5*, Fo_VPRI44637 *ex* onion and Fo_VPRI44638 *ex* onion also contained *CRX1*.

Fp_VPRI44629 *ex* garlic was the most pathogenic of the FP isolates to garlic and contained the pre-characterised putative effectors *SIX2*, *SIX13*, and *CRX2*. The remaining three FP isolates also contained these putative effectors with the exception of *SIX13*, which was absent, and instead had *SIX9*, which was not present in Fp_VPRI44629. *Fusarium oxysporum* isolates contained *SIX9*, *SIX13*, *C5*, *CRX1* and *CRX2*, while FP isolates had *SIX2*, *SIX9*, *SIX13*, *CRX1* and *CRX2* (Table 7). *SIX2* was not found in FO with garlic or onion as a host but was in FP. Interestingly, Fp_VPRI44629 *ex* garlic did not contain *SIX9*, which was present in the other three FP isolates, but did have *SIX13*, which was not identified in any other FP isolate in this study.

**Figure 2:**
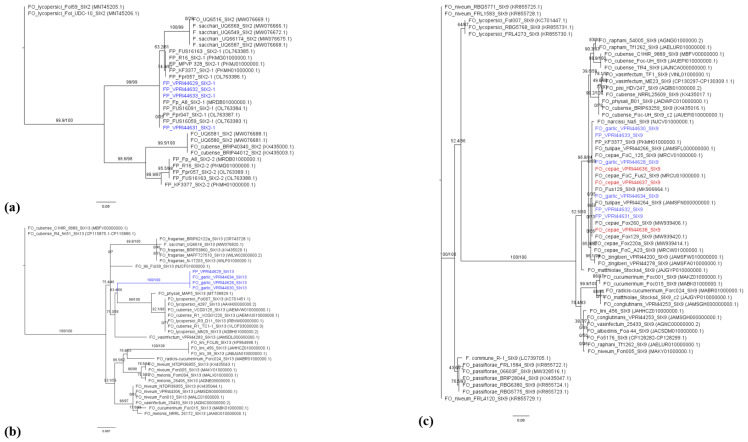
Maximum likelihood phylogenetic trees of pre-characterised putative effectors (**a**) *SIX2*, (**b**) *SIX9*, and (**c**) *SIX13*. The blue-coloured isolates indicate the FP and FO isolates *ex* garlic. The red-coloured isolates indicate the FO f. sp. *cepae* isolates *ex* onion from this study. The isolates in black are reference sequences from Supplementary Tables S2 and S3, and the GenBank accession numbers are in brackets.

**Figure 3:**
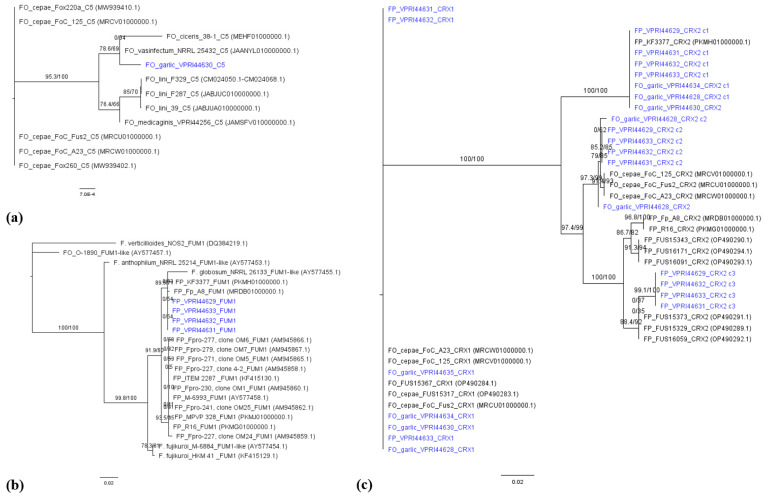
Maximum likelihood phylogenetic trees of genes (**a**) *C5*, (**b**) *FUM1*, (**c**) *CRX1/CRX2*. The blue-coloured isolates indicate the FP and FO isolates ex garlic. The isolates in black are reference sequences from Supplementary Tables S2 and S3. The GenBank accession numbers are in brackets. In (**c**) the *CRX1* and *CRX2* tree, the c1 indicates copy 1, c2 indicates copy 2 and c3 indicates copy 3 of the *CRX2* gene.

Gene trees were made for *SIX9*, *SIX13*, *SIX2*, *C5*, *CRX1*, *CRX2* and *FUM1* with sequences from the FP and FO isolates from garlic as well as the corresponding sequences of FO genomes available in GenBank (Figure 2 and Figure 3). A homologue of the putative effector *SIX2*, known as *SIX2-1*, was identified in all four FP isolates from garlic which shared 100% sequence identity with each other and with other FP isolates Fp_A8 *ex* onion, R_16 *ex* onion, FUS16091, FUS16059 and Fpr047. The *SIX2* homologue *SIX2-2* was not determined to be present in the FP isolates from garlic, but BLAST results found it was present in other FP isolates.

Putative effector *SIX13* was present in Fp_VPRI44629 *ex* garlic, Fo_VPRI44628, Fo_VPRI44630 *ex* garlic, and Fo_VPRI44634 *ex* garlic which shared 100% sequence with each other and other *Fusarium* isolates from garlic. The highest identity of the *SIX13* sequence of *Fusarium* isolates from garlic with any other ff. spp. was 96.25% in FO f. sp. *vasinfectum* and FO f. sp. *niveum*. The *SIX13* sequence of FO isolates from garlic had 86–95.7% identity with FO f. sp. *lycopersici* isolates.

Putative effector *SIX9* was identified in six of the eight isolates derived from garlic in this study: Fo_VPRI44628, Fo_VPRI44630, Fo_VPRI44634, Fp_VPRI44631, Fp_VPRI44632, and Fp_VPRI44633. The *SIX9* sequences of these isolates were 100% identical to each other and to FP KF3377 *ex* garlic, FO f. sp. *tulipae* isolates VPRI44266 *ex* tulip and VPRI44264 *ex* tulip, and FOC isolates FoC_Fus2 *ex* onion, FoC_125 *ex* onion, Fo_VPRI44636 *ex* onion, Fo_VPRI44637 *ex* onion, and Fo_VPRI44638 *ex* onion. These isolates also had high identity (99.5–99.8%) of the *SIX9* sequence with other FOC isolates included in this study but only 63.1% identity with FO f. sp. *lycopersici* isolates.

For the putative effector *C5* there was high sequence identity for all isolates included in this study; the FOC isolates shared 100% identity with each other, and Fo_VPRI44630 *ex* garlic had 99.41% identity with the FOC isolates. The sequence of *C5* for Fo_VPRI44630 *ex* garlic shared the highest identity with FO f. sp. *vasinfectum* NRLL25432 with 99.9%.

Putative effector *CRX1* was found in all the isolates from garlic in this study except Fp_VPRI44629. The *CRX1* genes from these isolates as well as others from GenBank were added to the gene tree. The FP and FO isolates from garlic and FOC isolates from onion had 100% identity. *CRX1* genes had 86% identity to *CRX2* genes in their respective isolates. For *CRX2*, there were three copies found in the four garlic FP isolates; there were two copies in Fo_VPRI44634 *ex* garlic; one each in Fo_VPRI44628 *ex* garlic and Fo_VPRI44630 *ex* garlic; and no *CRX2* homologue in Fo_VPRI44635 *ex* garlic.

The mycotoxin *FUM1* gene was identified in all four FP isolates, but not from the FO isolates in this study. The FP isolates from garlic shared 100% sequence identity of *FUM1* with each other and 99.8% identity with FP isolate Fp_A8 *ex* onion, as well as 99.6% identity with FP isolates R_16 *ex* onion and KF3377 *ex* garlic.

## 4. Discussion

### 4.1. Pathogenicity and Pre-Characterised Putative Effectors Identified in F. proliferatum and F. oxysporum Isolated from Allium spp.

This research is the first identification of the potential genes related to pathogenicity in FP and FO isolated from garlic. In particular, this research reports new information on the *SIX* genes and putative effectors identified in FP and FO. The results of sequencing and presence/absence of pathogenicity-associated genes were also compared to functionality through the pathogenicity assays. The pathogenicity-associated genes found in FO *ex* garlic were compared to the pathogenicity-associated gene profile of FOC *ex* onion. Notably, the putative effector profile of FP and FO *ex* garlic were more thoroughly examined using both WGS and PCRs compared to FO f. sp. *cepae ex* onion, which was investigated using PCRs only in this present study.

The seedling and storage assays used in this study measure partially different pathogenicity mechanisms of fungal isolates [30], and thus it is important to explore both pathogenicity assays. Generally, seedlings are more susceptible to disease infection than mature plants, possibly due to improved resistance over time [46]. There is high variability in the virulence of *Fusarium* isolates in alliums, and the virulence is dependent on the plant species, cultivar and crop stage [47]. In this study, within the limited number of isolates evaluated, the *Fusarium* isolates nonetheless had highly variable pathogenicity on the allium species (garlic, shallot and onion).

Several putative effectors for pathogenicity in *Fusarium* species are identified in the literature. This particularly includes the *SIX* genes that encode secreted effector proteins containing cysteine residues and contribute to FO pathogenicity and virulence [48]. In total, 14 *SIX* genes have been identified, and the presence or absence of these and the sequence variation of these genes may be used to determine ff. spp. or races of FO [48]. In the present study, the putative effectors *SIX9*, *CRX1* and *CRX2* were identified in FP, FO *ex* garlic and FOC *ex* onion. Comparatively, *SIX13*, and *C5*, *CRX1* and *CRX2* were identified in FO isolates *ex* garlic, and putative effectors *SIX2*, *SIX9*, *SIX13*, *CRX1* and *CRX2* were identified in FP isolates. Additionally, in FOC, the putative effector genes *SIX3*, *SIX5*, *SIX7*, *SIX9*, *SIX10*, *SIX12*, *SIX14*, *C5*, *CRX1* and *CRX2* were identified [12,14]. The putative effectors *C5*, *CRX1* and *CRX2* were first identified in FOC [12]. In research conducted by both Taylor et al. [12] and Haapalainen et al. [14,15], the presence of these putative effector genes was strongly correlated to higher pathogenicity of isolates and was expressed in onion *in planta* [12,14].

In Experiment 1 (pathogenicity of FP and FO *ex* garlic on garlic in-storage), Fo_VPRI44630, Fp_VPRI44629, Fp_VPRI44631, and Fp_VPRI44633 were significantly more pathogenic than Fo_VPRI44635 *ex* garlic. The least pathogenic isolate in this experiment, Fo_VPRI44635, only contained one putative effector (CRX1), while the isolates that were significantly more pathogenic on garlic in storage contained 3–5 putative effectors from *SIX2*, *SIX9*, *SIX13*, *CRX1*, *CRX2* and *C5*. The differing virulence of isolates on garlic and other alliums in this study may be attributed to the different putative effector profiles.

In Experiment 3B, which examined the pathogenicity of FO *ex* garlic and FOC *ex* onion on garlic in storage, Fo_VPRI44630 *ex* garlic was the most pathogenic isolate and contained *SIX9*, *SIX13*, *C5*, *CRX1*, and *CRX2*. Fo_VPRI44630 *ex* garlic was more pathogenic on garlic than the FOC isolate Fo_VPRI44637 *ex* onion which contained *SIX3*, *SIX5*, *SIX7*, *SIX9*, *SIX10*, *SIX12*, *SIX14*, *C5*, *CRX1* and *CRX2*. This result indicates the potential importance of specific effectors for host range and specificity and potential redundancy of effectors on specific hosts. Fo_VPRI44630 *ex* garlic was also significantly more pathogenic than Fo_VPRI44634 *ex* garlic, which contained *SIX9*, *SIX13*, *CRX1*, and *CRX2*, and Fo_VPRI44635 *ex* garlic, which only contained putative effector *CRX1*. In Experiment 3A, Fo_VPRI44630 *ex* garlic was the most pathogenic isolate on garlic. The FOC isolate Fo_VPRI44638 *ex* onion, with 9–11 putative effectors, was more pathogenic than Fo_VPRI44628 *ex* garlic, with *SIX9*, *SIX13*, *CRX1*, and *CRX2*, and Fo_VPRI44635 *ex* garlic, with only *CRX1*.

Across five experiments on garlic (in-storage and seedlings), Fo_VPRI44630 *ex* garlic was consistently the most pathogenic isolate, and in Experiments 3–7, FOC isolate Fo_VPRI44637 *ex* onion was the next most strongly pathogenic isolate on garlic. Although the difference between disease ratings for the two isolates was not significant, the consistency of Fo_VPRI44630 *ex* garlic having the highest pathogenicity on garlic across experiments appears important. Both isolates Fo_VPRI44630 *ex* garlic and Fo_VPRI44637 *ex* onion contained the *SIX9*, *C5*, *CRX1*, and *CRX2* putative effectors, while Fo_VPRI44630 *ex* garlic also had *SIX13* which was not present in FOC isolate Fo_VPRI44637 *ex* onion. Despite the presence of several additional putative effector genes in Fo_VPRI44637 *ex* onion, including *SIX3*, *SIX5*, *SIX7*, *SIX10*, *SIX12*, and *SIX14*, the pathogenicity was not as strong as that for Fo_VPRI44630 *ex* garlic. This suggests that the role of the putative effector *SIX13* in the pathogenicity of FO in garlic should be explored. However, Fo_VPRI44634 also contained the *SIX13* putative effector gene but was not as pathogenic as Fo_VPRI44630 *ex* garlic, suggesting combinations of these specific putative effectors are potentially contributing to the pathogenicity of different isolates on specific hosts. The highly virulent isolate Fo_VPRI44630 *ex* garlic contained putative effector *C5,* which was not present in the other FO *ex* garlic isolates in this study. However, as discussed above, *C5* was also present in Fo_VPRI44637 *ex* onion. Therefore, the function of specific putative effectors, including *SIX13* and *C5*, in the pathogenicity of alliums should be investigated, especially in the context of identifying the role of specific putative effectors in host specificity and effector redundancy in other hosts. Future research on the expression of these genes *in planta* in garlic and onion and a wider analysis of the putative effector profile of *Fusarium* isolates from alliums from diverse locations would help examine the functional role of putative effectors in pathogenicity.

Interestingly, Fo_VPRI44635 *ex* garlic was the least pathogenic isolate in all pathogenicity experiments and contained the least putative effectors of all isolates. Despite appearing only mildly pathogenic on garlic cloves and shallot and onion bulbs in storage, this isolate nonetheless severely decreased germination of onion seeds. A similar finding was observed by Haapalainen et al. [15], where onion seed germination was severely reduced by mildly pathogenic isolates that did not contain any *SIX* genes. In the same study, FO isolates that did not contain *SIX* genes but did contain *CRX2* were significantly more pathogenic than isolates that were missing *CRX2* [15]. Furthermore, *F. oxysporum* isolates lacking the known putative effectors have caused significant reductions in seedling emergence [15].

In this study, seven of the eight isolates cultured from garlic were from visibly diseased garlic cloves, while one isolate Fp_VPRI44629 *ex* garlic was isolated from an apparently healthy clove with no visible disease symptoms. This isolate caused moderate to severe disease symptoms in garlic and contained the putative effectors *SIX2*, *SIX13*, *CRX1* and *CRX2*. *Fusarium proliferatum* and FO have often been isolated from symptomless garlic cloves and onion bulbs [8,9,30]. Latent infections of *Fusarium* in garlic bulbs at harvest are common, where symptomless bulbs or cloves develop fusarium dry rot symptoms after 2 months of storage [9]. This is a major issue in garlic production as garlic bulbs are stored for up to 6 to 8 months prior to replanting in the subsequent season [9], and stored product contributes to continued market supply.

### 4.2. Sequences of Pre-Characterised Putative Effectors in Fusarium spp. and the Importance of the Identification and Sequence of These Genes

Gene trees of the specific putative effectors found in FO *ex* garlic were produced to examine the phylogeny of the putative effector genes. The sequences for *SIX13*, *SIX9* and *CRX1* had high homogeneity among FO *ex* garlic isolates in this study. For *SIX9*, FO *ex* garlic isolates had 100% identity to FO f. sp. *tulipae* and FO f. sp. *cepae* isolates and formed a well-supported clade with these isolates. Thus, the sequence of *SIX9* in FO *ex* garlic isolates was not specific to its own f. sp. or race, and it did not separate from other ff. spp. based on this sequence. There has been limited research into the pathogenicity genes identified in FP infecting alliums, but the publicly available genome for FP FF3377 *ex* garlic contained putative effectors *SIX9*, *SIX2-1*, *SIX2-2*, and *CRX2*. This is an interesting finding since *SIX9* has not previously been identified in FP, and there was 100% sequence identity with *Fusarium* spp. from alliums. Taylor, Vágány [12] demonstrated *SIX9* as being expressed *in planta* in onions, but there is no research into the function of *SIX9* during infection [49].

The sequences of *SIX13* from FO *ex* garlic and FP *ex* garlic isolates had 100% identity with each other, and the *SIX13* sequence was distinct from any other ff. spp. The *Fusarium* isolates from garlic formed their own well-supported clade, with the closest identity with other ff. spp. being 96.3% in FO f. sp. *vasinfectum* and FO f. sp. *niveum*. Horizontal gene transfer between species has been suggested to have occurred between *Fusarium* species for other *SIX* genes [50,51]. It is possible that *SIX13* and *SIX9* were acquired in FP *ex* garlic through horizontal gene transfer from FO *ex* garlic, since the sequence had high homology between the different species, and thus not vertically transferred among each other since it is not a process for genetic transfer between different species.

A *SIX2* homologue (*SIX2-1*) was identified in all four FP isolates from garlic, which had high sequence homogeny and was not present in FO isolates in this study. Haapalainen, Kuivainen [15] found that all 15 FP isolates in their study carried *SIX2-1* and determined that it likely has an important function in FP isolates and should be investigated. The effector *SIX2* has been shown to increase the pathogenicity of FOL in tomato seedlings [15,52]. A *SIX2* homologue has been widely identified in the *F. fujikuroi* species complex, which includes FP, and the *SIX2* gene was likely vertically inherited since the phylogeny of the *SIX2* gene was similar to the phylogenetic species tree in Figure 1 [50].

Of the *Fusarium* isolates from garlic, the *C5* putative effector was only present in Fo_VPRI44630 *ex* garlic determined by a BLAST+ search of the genomes and confirmed using PCR. The putative effector *CRX1* was identified in three of the isolates of FP and four of the isolates of FO *ex* garlic, and these isolates shared 100% identity of the *CRX1* gene sequence. Although isolate Fp_VPRI44629 *ex* garlic did not contain the *CRX1* gene, it had three copies of *CRX2*. All of the FP isolates and Fo_VPRI44634 from garlic had multiple copies of *CRX2* since there were multiple distinct and complete sequence hits in the genomes identified using a BLAST+ search. In support of this finding, previous research examining *Fusarium* isolates in onion has shown that FO and FP isolates frequently contain multiple copies of *CRX2* [12,15]. The gene tree for *CRX1* and *CRX2* showed clear separation of *CRX1* and *CRX2* sequences, which has also been demonstrated previously by Taylor, Vágány [12].

The gene *FUM1*, a component of the *FUM* gene cluster and known for its role in producing the mycotoxin fumonisin, was present in all four FP isolates but was not identified in any of the FO isolates. *Fusarium proliferatum* isolates in this study shared 100% identity of the *FUM1* gene sequence. The presence of *FUM1* gene is correlated to fumonisin production [53], and fumonisins are known to produce a toxic effect on plants, animals and humans [54,55,56]. Therefore, the presence of *FUM1* may be contributing to the pathogenicity of FP on garlic, highlighting the importance of further research into the risk to food safety that FP infection in alliums poses.

The identification of the pathogenicity-associated genes, particularly the *SIX* genes, is of significant importance since effectors in *Fusarium* spp. may correlate to different resistance genes in the host. However, further research on a larger number of isolates from diversified locations from both onion and garlic plants would be required to support this concept. In plant diseases caused by FO, host resistance is the most effective management strategy [57,58]. In tomato, *Fusarium oxysporum* f. sp. *lycopersici* (FOL) races are divided by their ability to overcome resistance-related genes, which implied a gene-for-gene model for the host–pathogen system between effectors in FOL and the tomato I resistance genes to FOL [58]. The tomato I (immunity) genes have been identified as important for conferring resistance to Fusarium wilt [58]. Importantly, some of the resistance genes identified in tomato have been directly related to the putative effector *SIX* genes present in FOL [58,59], therefore highlighting this research as an important initial step in characterising the pathogenicity-related genes in FO and FP that could help identify host resistance for garlic in the future.

### 4.3. Host-Associated Differentiation of Pathogenicity in F. oxysporum Isolated from Alliums

The results of this study suggest a difference in host-associated pathogenicity; therefore, a discussion on f. sp. designation in pathogenic FO from *Allium* spp. is warranted. Notwithstanding, this research alone does not indicate strong evidence of a robust f. sp. or race designation of FO from *Allium* spp., and further validation is required. Within the species complex *Fusarium oxysporum* there is also f. sp. classification, which is grouped together based on host specificity [10]. Grouping f. sp. is challenging since standard molecular methods, including DNA fingerprinting and multilocus genotyping with housekeeping genes, cannot be used to successfully distinguish ff. spp. or races [19,20]. This was confirmed in this study where the clades containing the FO *ex* garlic isolates in the multilocus maximum likelihood tree (Figure 1) also contained other ff. spp. [12]. However, there are multiple instances where the *SIX* gene profile and sequences have been used to discern ff. spp. and races [19,20].

Currently, there are an estimated 106 characterised ff. spp. of FO, including f. sp. *cepae* that infects onion and other alliums [10,12,13,14,15] and f. sp. *garlic* that infects garlic [5,7,10]. However, previous research has assumed that FO infecting garlic was f. sp. *cepae* [16,17,18]. Within ff. spp. there are also races, but to date neither f. sp. *cepae* nor f. sp. *garlic* have been identified as having races [10]. In the present study, it was identified that FO isolates *ex* onion were pathogenic to garlic, while the results of Matuo et al. [7] found FOC infected onion seedlings but not garlic cloves, and FO f. sp. *garlic* infected garlic cloves but not onion seedlings. Since the research of Matuo et al. [7] was conducted ~40 years ago, molecular analysis and gene sequencing for pathogenicity genes (e.g., *SIX* genes) was not conducted. There have also been enormous developments in the understanding of pathogenicity genes due to the development of advanced sequencing technologies, and during that time, pathogens may have changed and evolved; thus, having a difference in pathogenicity results is not surprising. Consistent with the present study findings, Dugan et al. [60] demonstrated that FOC and FO *ex* garlic were both able to cause disease symptoms in both garlic and onion plants. Additionally, Dedecan et al. [5] identified FO f. sp. *garlic* based on the pathogenicity results of FO isolates from garlic, which caused disease symptoms in garlic but not onion seedlings, but the authors only used two isolates. This result is similar to the pathogenicity results of FO isolates from garlic on shallot bulbs since two of the isolates in this research were not significantly different from control plants. Dedecan et al. [5] described the pathogenicity test methods for garlic well, but the methods used for onion were not defined. However,, in agreement with the results from this study, research by Dedecan et al. [5] has demonstrated that FOC and FO *ex* garlic were both able to cause disease symptoms in both garlic and onion plants [60].

The results for pathogenicity tests showed a distinct difference in virulence between FO isolates on allium species. *Fusarium oxysporum ex* garlic mostly caused disease symptoms or just mild symptoms in shallot and only mild to moderate symptoms in onion, while FO from onion caused severe disease symptoms in shallot, garlic and onion. This indicates that the FO isolates *ex* garlic have a more specific virulence to garlic plants, while FO isolates *ex* onion can infect and cause disease more generally across *Allium* species. The difference in virulence and differing putative effector profiles between FO *ex* garlic and FO *ex* onion suggest there is host-associated differentiation of pathogenicity. Therefore, further investigation of the f. sp. or race classification of pathogenic FO isolates from *Allium* spp. is required, using a larger number of isolates from diversified locations from both onion and garlic plants to support this concept. Future research into the distinction between f. sp. or race could be important for future diagnostics and management of FO in alliums.

## Figures and Tables

**Table 1:** Details for each pathogenicity experiment (Exp.) including host plant species, *Fusarium* isolate species, inoculation method, number of replicates, incubation or growth conditions, assessment metric and statistical analysis.

Exp.	Host Species (Cultivar) & Plant Part/Stage	Isolate Set (Treatment + Control)	Inoculation Method (Inoculum = 10^6^ Conidia mL^−1^)	Replicates per Treatment	Incubation/Growth Conditions	Assessment Metric & Timing	Statistical Analysis
Pathogenicity of FP *ex* garlic and FO *ex* garlic isolates on garlic							
**1**	Garlic clove (storage)	4 FO garlic + 4 FP garlic + water	Injection 100 µL at 1–1.5 cm depth	15 cloves	Dark, 24 °C, 16 days, individual zip-bags	Disease rating 0–4 on cut clove (Supplementary Figure S1)	Ordinal logistic regression
**2**	Garlic seedling (from cloves)	Same isolate set as Exp. 1	Immersion of cloves in 150 mL suspension, 1 h, 80 rpm → planted	12 plants	24 °C, 14 h photoperiod, 3 weeks	Disease rating 0–4 on plant/clove (Supplementary Figure S2)	Ordinal logistic regression
Pathogenicity of FO *ex* garlic and FO *ex* onion isolates on garlic, onion and shallot							
**3A**	Garlic clove ‘Argentina’ (storage)	4 FO garlic + 3 FOC onion + water	Injection 100 µL	12 cloves	Dark, 24 °C, 16 days	Disease rating 0–4 (Supplementary Figure S3)	Ordinal logistic regression
**3B**	Garlic clove ‘Glenlarge’ (storage)	Same isolate set as Exp. 3A	Injection 100 µL	15 cloves	Dark, 24 °C, 14 days	Disease rating 0–4 (Supplementary Figure S3)	Ordinal logistic regression
**4**	Garlic seedling	Same isolate set as Exp. 3A	Immersion of cloves as in Exp. 2	12 plants	24 °C, 14 h photoperiod, 3 weeks	Disease rating 0–4 (Supplementary Figure S4)	Ordinal logistic regression
**5**	Shallot bulb ‘Tuktuk’ (storage)	Same isolate set as Exp. 3A	Injection 100 µL	10 bulbs	Bench, 24 °C, open containers, 16 days	Disease rating 0–4 (Supplementary Figure S5)	Ordinal logistic regression
**6**	Onion bulb ‘ONAUS21.17630’ (storage)	Same isolate set as Exp. 3A	Injection 100 µL	10 bulbs	Bench, 20–24 °C, 17 days	Disease rating 0–4 (Supplementary Figure S6)	Ordinal logistic regression
**7**	Onion seed/seedling ‘ONAUS21.17630’	Same isolate set as Exp. 3A	Immersion of seeds in 50 mL suspension, 1 h, 80 rpm → planted	4 pots × 12 seeds (48)	24 °C, 12 h photoperiod, 6 weeks	Cumulative germination & weekly mortality	Logistic regression

**Table 2:** Primers, primer sequences, annealing temperatures of PCRs and references used in this study.

Primer Name	Sequence	Annealing Temperature (°C)	Reference
EF1	ATGGGTAAGGARGACAAGAC	64	[26]
EF2	GGARGTACCAGTSATCATG		
EF3	GTAAGGAGGASAAGACTCACC	NA—Sequencing primers	[27]
EF22T	AGGAACCCTTACCGAGCTC		
FprSIX2cF	TCCTCCAGGCTACGTTCACAAG	65	[15]
FprSIX2cR	AAACCAGCCATTATAGCCGTCG		
FprSIX2-1F	GTTCTTTAAGCATGCGTTAGTC	60	[15]
FprSIX2-1R	GCTTTTCCATATTACAATGCCTCC		
FprSIX2-2F	ACGACTATCTATACACAACACG	60	[15]
FprSIX2-2R	TTTCTCGTCGTAGCTTTTAGAC		
CRX2-FPF	CCAGTGCATTGGTTTGAGACGTT	63	[15]
CRX2-FPR	ATGCGCTCGCTTTCTATGTATCTG		
FUM1P2-F	CCCCCATCATCCCGAGTAT	63	[15]
FUM1P2-R	TGGGTCCGATAGTGATTTGTCA		
CRX1-F	CACCATCTGTCTACATAAGGCCGCCC	69	[12]
CRX1-R	AAAGTTCAAGGACCGGACCGCCG		
SIX1F	GTATCCCTCCGGATTTTGAGC	59	[19]
SIX1R	AATAGAGCCTGCAAAGCATG		
SIX2F	CAACGCCGTTTGAATAAGCA	59	[19]
SIX2R	TCTATCCGCTTTCTTCTCTC		
SIX3F	CCAGCCAGAAGGCCAGTTT	59	[19]
SIX3R	GGCAATTAACCACTCTGCC		
SIX4F	TCAGGCTTCACTTAGCATAC	59	[19]
SIX4R	GCCGACCGAAAAACCCTAA		
SIX5F	ACACGCTCTACTACTCTTCA	59	[19]
SIX5R	GAAAACCTCAACGCGGCAAA		
SIX6F	CTCTCCTGAACCATCAACTT	59	[19]
SIX6R	CAAGACCAGGTGTAGGCATT		
SIX7F	CATCTTTTCGCCGACTTGGT	59	[19]
SIX7R	CTTAGCACCCTTGAGTAACT		
SIX8F	TCGCCTGCATAACAGGTGCCG	59	[40]
SIX8R	TTGTGTAGAAACTGGACAGTCGATGC		
FOCSIX9F	GGCCCAGCCCTAGTCTAACTCC	67	[12]
FOCSIX9R	AACTTAACATGCTGGCCGTCAATCG		
SIX10F	GTTAGCAACTGCGAGACACTAGAA	65	[12]
SIX10R	AGCAACTTCCTTCCTCTTACTAGC		
SIX11F	ATTCCGGCTTCGGGTCTCGTTTAC	61	[12]
SIX11R	GAGAGCCTTTTTGGTTGATTGTAT		
SIX12F	CTAACGAAGTGAAAAGAAGTCCTC	61	[12]
SIX12R	GCCTCGCTGGCAAGTATTTGTT61		
SIX13F	CCTTCATCATCGACAGTACAACG	61	[12]
SIX13R	ATCAAACCCGTAACTCAGCTCC		
FOCSIX14F	ACAACACCGCGACGCTAAAAAT	61	[12]
FOCSIX14R	GCACACTCAGTGCGACAAGTTC		
C5F	AGAGTGTGAAGTGAGGACGAGGGA	63	[12]
C5R	CTACGTTCGCCTCACTCATTGCCT		
CRX1F	CACCATCTGTCTACATAAGGCCGCCC	69	[12]
CRX1R	AAAGTTCAAGGACCGGACCGCCG		
CRX2F	TTAGTCGCACATCTACCATCACTG	58	[12]
CRX2R	GGAGTCGATCTAACTTCAGG		

**Table 3:** Assembly and annotation statistics for *Fusarium oxysporum* isolates *ex* garlic Fo_VPRI44630, Fo_VPRI44634, Fo_VPRI44635 and Fo_VPRI44628 and *Fusarium proliferatum* isolates *ex* garlic Fp_VPRI44629, Fp_VPRI44631, Fp_VPRI44632 and Fp_VPRI44633.

Sample	Fp_VPRI44629	Fp_VPRI44631	Fp_VPRI44632	Fp_VPRI44633	Fo_VPRI44630	Fo_VPRI44634	Fo_VPRI44635	Fo_VPRI44628
GenBank Accession	JBLWFC000000000	JBLWFB000000000	JBLWFA000000000	JBLWEZ000000000	JBKOPR000000000	JBLWFF000000000	JBLWFE000000000	JBLWFD000000000
Number of contigs	149	211	211	153	210	153	24	160
Total length (bp)	50,031,492	48,676,132	48,638,985	48,991,131	54,077,911	54,463,706	47,344,689	53,794,114
Mean contig length (bp)	335,781.8	230,692.6	230,516.5	320,203.5	257,513.9	355,971.9	1,972,695	336,213.2
N50 (bp)	2,848,431	3,531,743	3,540,231	3,546,590	3,592,903	4,252,426	4,501,934	4,399,489
Largest contig (bp)	5,827,024	7,103,470	7,106,942	7,095,604	6,196,020	6,447,342	6,411,834	6,586,364
Mean coverage (%)	75	111	143	121	101	118	126	119
GC content (%)	48.38	48.32	48.32	48.34	48	47.67	47.67	47.93
Number of protein-coding genes	16,937	16,681	16,590	16,767	16,714	17,362	15,778	17,443
mRNA	18,166	17,952	17,975	18,089	17,454	18,659	17,076	18,659
tRNA	278	286	283	280	306	309	300	296
Av. gene length (bp)	1825.83	1845.81	1856.39	1868.51	1671.95	1782.02	1809.03	1776.9
Total exons	74,731	74,953	76,375	77,047	62,318	74,463	69,977	74,363
Avg. protein length (bp)	475.17	476.68	479.16	476.42	467.13	478.85	479.36	476.02

**Table 4:** Mean disease rating (DR) results for pathogenicity tests of four *Fusarium proliferatum* and four *F. oxysporum* isolates *ex* garlic for Experiment (Expt) 1 and 2. Each column includes the mean ± SEM, and the different letters in each column indicate significant differences between treatments.

Isolate	Species	Garlic Clove in-Storage (Expt 1)	Garlic Seedling (Expt 2)
Control		0.3 ± 0.19 ^a^	0.1 ± 0.09 ^a^
Fp_VPRI44629	FP	2.6 ± 0.19 ^cd^	1.7 ± 0.37 ^b^
Fp_VPRI44631	FP	2.5 ± 0.15 ^cd^	1.2 ± 0.22 ^b^
Fp_VPRI44632	FP	2.1 ± 0.15 ^bcd^	1.2 ± 0.28 ^b^
Fp_VPRI44633	FP	2.3 ± 0.19 ^cd^	1.7 ± 0.30 ^b^
Fo_VPRI44630	FO	3.5 ± 0.19 ^d^	2.8 ± 0.17 ^b^
Fo_VPRI44634	FO	1.9 ± 0.19 ^bc^	1.9 ± 0.32 ^b^
Fo_VPRI44635	FO	0.9 ± 0.19 ^b^	1.1 ± 0.33 ^b^
Fo_VPRI44628	FO	1.9 ± 0.19 ^bc^	2.2 ± 0.33 ^b^

**Table 5:** Mean disease rating (DR) results for pathogenicity tests of four FO isolates from garlic and three FOC isolates from onion on garlic cloves (‘Argentina’ Experiment (Exp.) 3A and ‘Glenlarge’ Experiment 3B), garlic seedling (‘Glen-large’ Experiment 4), shallot bulb (‘Tuk Tuk’ Experiment 5) and onion bulb (Experiment 6). Each column includes the mean ± SEM, and the different letters in each column indicate significant differences between treatments.

Isolate	Isolate Origin Host	Exp. 3A	Exp. 3B	Exp. 4	Exp. 5	Exp. 6
Control		0.1 ± 0.08 ^a^	0.3 ± 0.16 ^a^	0.7 ± 0.28 ^a^	0.2 ± 0.13 ^a^	0.1 ± 0.10 ^a^
Fo_VPRI44630	Garlic	3.7 ± 0.14 ^c^	3.8 ± 0.14 ^d^	2.7 ± 0.40 ^b^	0.7 ± 0.15 ^b^	1.8 ± 0.25 ^bc^
Fo_VPRI44634	Garlic	2.8 ± 0.18 ^bc^	1.9 ± 0.12 ^bc^	2.3 ± 0.43 ^b^	0.5 ± 0.17 ^ab^	0.8 ± 0.25 ^b^
Fo_VPRI44635	Garlic	1.2 ± 0.30 ^b^	1.2 ± 0.17 ^b^	1.3 ± 0.28 ^ab^	1.1 ± 0.18 ^bc^	1.7 ± 0.21 ^bc^
Fo_VPRI44628	Garlic	2.3 ± 0.22 ^b^	2.3 ± 0.15 ^bcd^	2.2 ± 0.37 ^b^	0.6 ± 0.16 ^ab^	0.9 ± 0.23 ^b^
Fo_VPRI44636	Onion	3.2 ± 0.11 ^bc^	2.5 ± 0.26 ^bcd^	1.8 ± 0.44 ^b^	3.4 ± 0.16 ^d^	3.1 ± 0.28 ^c^
Fo_VPRI44637	Onion	3.1 ± 0.08 ^bc^	2.1 ± 0.18 ^bc^	2.3 ± 0.33 ^b^	2.4 ± 0.22 ^c^	2.4 ± 0.16 ^bc^
Fo_VPRI44638	Onion	3.3 ± 0.19 ^c^	3.0 ± 0.24 ^cd^	1.6 ± 0.34 ^b^	3.5 ± 0.17 ^d^	3.2 ± 0.20 ^c^

**Table 6:** Results of the proportion of emerged onion seedlings in Experiment 7. The different letters in each column indicate significant differences between treatments.

Isolate	Isolate Origin Host	Proportion of Emerged Onion Seedlings
Control		0.53 ± 0.07 ^a^
Fo_VPRI44630	Garlic	0.08 ± 0.05 ^bc^
Fo_VPRI44634	Garlic	0.08 ± 0.03 ^bc^
Fo_VPRI44635	Garlic	0.19 ± 0.02 ^b^
Fo_VPRI44628	Garlic	0.10 ± 0.06 ^bc^
Fo_VPRI44636	Onion	0.08 ± 0.03 ^bc^
Fo_VPRI44637	Onion	0.04 ± 0.02 ^c^
Fo_VPRI44638	Onion	0.10 ± 0.05 ^bc^

**Table 7:** Whole-genome sequencing and PCR detection of known putative effectors (pathogenicity-related genes) of *Fusarium* spp. The isolates highlighted green are FP, the isolates highlighted purple are FO *ex* garlic, the isolates highlighted blue are FOC *ex* onion, and the isolate highlighted orange is FO f. sp. *lycopersici*. The negative control consisted of nuclease-free water.

	*SIX1*	*SIX2*	*SIX3*	*SIX4*	*SIX5*	*SIX6*	*SIX7*	*SIX8*	*SIX9*	*SIX10*	*SIX11*	*SIX12*	*SIX13*	*SIX14*	*C5*	*CRX1*	*CRX2*
Negative Control	−	−	−	−	−	−	−	−	−	−	−	−	−	−	−	−	−
Fp_VPRI44629	−	+	−	−	−	−	−	−	−	−	−	−	+	−	−	−	+
Fp_VPRI44631	−	+	−	−	−	−	−	−	+	−	−	−	−	−	−	+	+
Fp_VPRI44632	−	+	−	−	−	−	−	−	+	−	−	−	−	−	−	+	+
Fp_VPRI44633	−	+	−	−	−	−	−	−	+	−	−	−	−	−	−	+	+
Fo_VPRI44630	−	−	−	−	−	−	−	−	+	−	−	−	+	−	+	+	+
Fo_VPRI44634	−	−	−	−	−	−	−	−	+	−	−	−	+	−	−	+	+
Fo_VPRI44635	−	−	−	−	−	−	−	−	−	−	−	−	−	−	−	+	−
Fo_VPRI44628	−	−	−	−	−	−	−	−	+	−	−	−	+	−	−	+	+
Fo_VPRI44636	−	−	−	−	+	−	+	−	+	+	−	+	−	+	+	−	−
Fo_VPRI44637	−	−	+	−	+	−	+	−	+	+	−	+	−	+	+	+	−
Fo_VPRI44638	−	−	+	−	+	−	+	−	+	+	−	+	−	+	+	+	−
Foc_Fus2	−	−	+	−	+	−	+	−	+	+	−	+	−	−	+	+	+
Foc_125	−	−	+	−	+	−	+	−	+	+	−	+	−	+	+	+	+
Foc_A23	−	−	+	−	+	−	+	−	+	+	−	+	−	−	+	+	+
FOLR1	+	+	+	−	+	+	+	+	−	+	+	+	+	−	−	−	−

## Data Availability

The data presented in this study is openly available in NCBI GenBank at https://www.ncbi.nlm.nih.gov/, reference numbers JBLWFC000000000, JBLWFB000000000, JBLWFA000000000, JBLWEZ000000000, JBKOPR000000000, JBLWFF000000000, JBLWFE000000000, JBLWFD000000000.

## Supplementary Materials

The following supporting information can be downloaded at: https://www.mdpi.com/article/10.3390/jof12040264/s1. Refs. [61,62,63,64,65] were referenced in the supplementary text. Identification and Morphology of Fusarium isolates in this study. Figure S1. Experiment 1 results for pathogenicity of F. oxysporum and FP *ex* garlic on garlic cloves. Disease severity was assessed using a rating scale from 0 to 4. (a) DR 0—No disease symptoms. (b) DR 1—Mild symptoms included discolouration at the site of injection (up to 10% of the clove displaying discolouration). (c) DR 2—Moderate symptoms consisted of discolouration beyond the site of injection (~10–30% of clove having rot symptoms). (d) DR 3—Moderate to severe symptoms consisted of cloves with 30–50% of the clove having discolouration/rotting symptoms and severe symptoms. (e) DR 4—Highly severe disease symptoms consisted of 50–100% rotting of the clove. Figure S2. Experiment 3 disease rating results of FP and FO *ex* garlic in garlic seedlings. Disease severity was assessed using a rating scale from 0 to 4. (a) DR 0—No disease symptoms. (b) DR 1—Mild symptoms included up to 10% of the clove having discolouration and usually at the baseplate. (c) DR 2—Moderate symptoms consisted of ~10–30% of clove having rot symptoms. (d) DR 3—Moderate to severe symptoms consisted of cloves with 30–50% of the clove having discolouration/rotting symptoms. (e) DR 4—Highly severe disease symptoms consisted of 50–100% rotting of the clove. Figure S3. Disease ratings for Experiment 4 results for pathogenicity tests of FO *ex* garlic and FOC *ex* onion on garlic cloves. Disease severity was assessed using a rating scale from 0 to 4. (a) DR 0—No disease symptoms. (b) DR 1—Mild symptoms included discolouration at the site of injection (up to 10% of the clove displaying discolouration). (c) DR 2—Moderate symptoms consisted of discolouration beyond the site of injection (~10–30% of clove having rot symptoms). (d) DR 3—Moderate to severe symptoms consisted of cloves with 30–50% of the clove having discolouration/rotting symptoms and severe symptoms. (e) DR 4—Highly severe disease symptoms consisted of 50–100% rotting of the clove. Figure S4. Disease ratings for Experiment 5 results for pathogenicity tests of FO *ex* garlic and FOC *ex* onion on garlic seedlings. Disease severity was assessed using a rating scale from 0 to 4. (a) DR 0—No disease symptoms. (b) DR 1—Mild symptoms included up to 10% of the clove having discolouration and usually at the baseplate. (c) DR 2—Moderate symptoms consisted of ~10–30% of clove having rot symptoms. (d) DR 3—Moderate to severe symptoms consisted of cloves with 30–50% of the clove having discolouration/rotting symptoms. (e) DR 4—Highly severe disease symptoms consisted of 50–100% rotting of the clove. Figure S5. Experiment 6 results for disease ratings for pathogenicity tests of FO *ex* garlic and FOC *ex* onion on mature shallot bulbs. Disease severity was assessed using a rating scale from 0 to 4. (a) DR 0—No disease symptoms. (b) DR 1—Mild symptoms included discolouration at the site of injection (up to 10% of the bulb displaying discolouration). (c) DR 2—Moderate symptoms consisted of discolouration beyond the site of injection (~10–30% of bulb having rot symptoms). (d) DR 3—Moderate to severe symptoms consisted of bulbs with 30–50% of the clove having discolouration/rotting symptoms. (e) DR 4—Highly severe disease symptoms consisted of 50–100% rotting of the bulb. Figure S6. Experiment 7 results for disease ratings for pathogenicity tests of FO *ex* garlic and FOC *ex* onion on mature onion bulbs. Disease severity was assessed using a rating scale from 0 to 4. (a) DR 0—No disease symptoms. (b) DR 1—Mild symptoms included discolouration at the site of injection (up to 10% of the bulb displaying discolouration). (c) DR 2—Moderate symptoms consisted of discolouration beyond the site of injection (~10–30% of bulb having rot symptoms). (d) DR 3—Moderate to severe symptoms consisted of bulbs with 30–50% of the clove having discolouration/rotting symptoms. (e) DR 4—Highly severe disease symptoms consisted of 50–100% rotting of the bulb. Table S1. Identification and Morphology of *Fusarium* isolates in this study. Table S2. Reference sequences for BLASTn search of *Fusarium* genomes included in this study. Table S3. *Fusarium* spp. genomes downloaded from GenBank and used in phylogenetic analysis. Table S4. *SIX2*, *SIX9*, *SIX13*, *CRX1*, *CRX2*, *C5*, and *FUM1* sequence accessions downloaded from GenBank and used in phylogenetic analysis.

## Abbreviations

The following abbreviations are used in this manuscript:

DR	Disease rating
FP	*Fusarium proliferatum*
FO	*Fusarium oxysporum*
FOC	*Fusarium oxysporum* f. sp. *cepae*
f. sp.	*Formae specialis*
ff. spp.	*Formae speciales*
*ex*	From
*SIX*	Secreted in xylem
*TEF*	Translation elongation factor 1α
*RPB1*	DNA-directed RNA polymerase II largest subunit
*RPB2*	DNA-directed RNA polymerase II second largest subunit
*TUB*	Beta-tubulin
SARDI	South Australian Research and Development Institute
NCBI	National Center for Biotechnology Information
WGS	Whole-genome sequencing
