# Supplementary material for: Pathogenicity and Pre-Characterised Putative Effectors of Fusarium oxysporum and F. proliferatum in Garlic (Allium sativum) and Other Allium spp
Source: J Fungi (Basel). 2026 Apr 6;12(4):264. doi: 10.3390/jof12040264 (PMC13117426; doi:10.3390/jof12040264)
Supplement: Supplementary file 1 [file jof-12-00264-s001.zip › jof-4222676-supplementary.pdf]

Table S1. Identification and Morphology of *Fusarium* isolates in this study.

Isolate	GenBank Accession	Species (TEF)	Origin Host	Origin Location	Collection date	Collector	Origin Plant Status	Colour on ¼ PDA	Pigment distribution	Aerial growth appearance
Fp_VPRI44629	PQ867234	FP	Garlic	Gatton, QLD, Australia	24-02-2023	J. Harper	Healthy	Orange-pink	Radial	Cotton-wool like
Fp_VPRI44631	PQ867235	FP	Garlic	Gatton, QLD, Australia	24-02-2023	J. Harper	Diseased	Pink	Radial	Cotton-wool like
Fp_VPRI44632	PQ867236	FP	Garlic	Gatton, QLD, Australia	24-02-2023	J. Harper	Diseased	Pink	Radial	Cotton-wool like
Fp_VPRI44633	PQ867237	FP	Garlic	Gatton, QLD, Australia	24-02-2023	J. Harper	Diseased	Pink	Radial	Cotton-wool like
Fo_VPRI44630	PQ867230	FO	Garlic	Gatton, QLD, Australia	24-02-2023	J. Harper	Diseased	White	Solid	Flat
Fo_VPRI44634	PQ867231	FO	Garlic	Gatton, QLD, Australia	24-02-2023	J. Harper	Diseased	White	Solid	Cotton-wool like
Fo_VPRI44635	PQ867232	FO	Garlic	Gatton, QLD, Australia	24-02-2023	J. Harper	Diseased	White-purple	Radial	Powdery
Fo_VPRI44628	PQ867233	FO	Garlic	Gatton, QLD, Australia	24-02-2023	J. Harper	Diseased	White	Radial	Powdery
Fo_VPRI44636	PQ867240	FO	Onion	Narromine, NSW, Australia	NA	Enza Zaden	NA	White-purple	Radial	Cotton-wool like
Fo_VPRI44637	PQ867238	FO	Onion	Upper Southeast SA, Australia	19-11-2021	M. Rettke & C. Todd	NA	White-purple	Radial	Cotton-wool like
Fo_VPRI44638	PQ867239	FO	Onion	Darling Downs, QLD, Australia	18-1-2022	M. Rettke & C. Todd	NA	White-purple	Radial	Flat

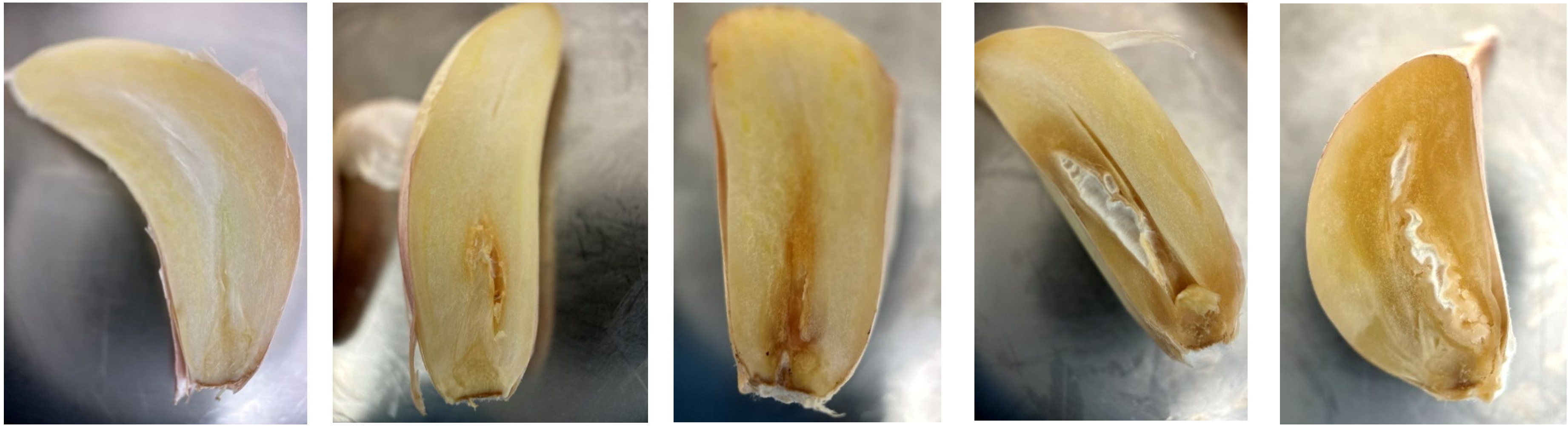

(a) (b) (c) (d) (e)

Figure S1. Experiment 1 results for pathogenicity of F. oxysporum and FP *ex* garlic on garlic cloves. Disease severity was assessed using a rating scale from 0 to 4. **(a)** DR 0 – No disease symptoms. **(b)** DR 1 – Mild symptoms included discolouration at the site of injection (up to 10% of the clove displaying discolouration). **(c)** DR 2 – Moderate symptoms consisted of discolouration beyond the site of injection (~10-30% of clove having rot symptoms). **(d)** DR 3 – Moderate to severe symptoms consisted of cloves with 30-50% of the clove having discolouration/rotting symptoms and severe symptoms. **(e)** DR 4 – Highly severe disease symptoms consisted of 50-100% rotting of the clove.

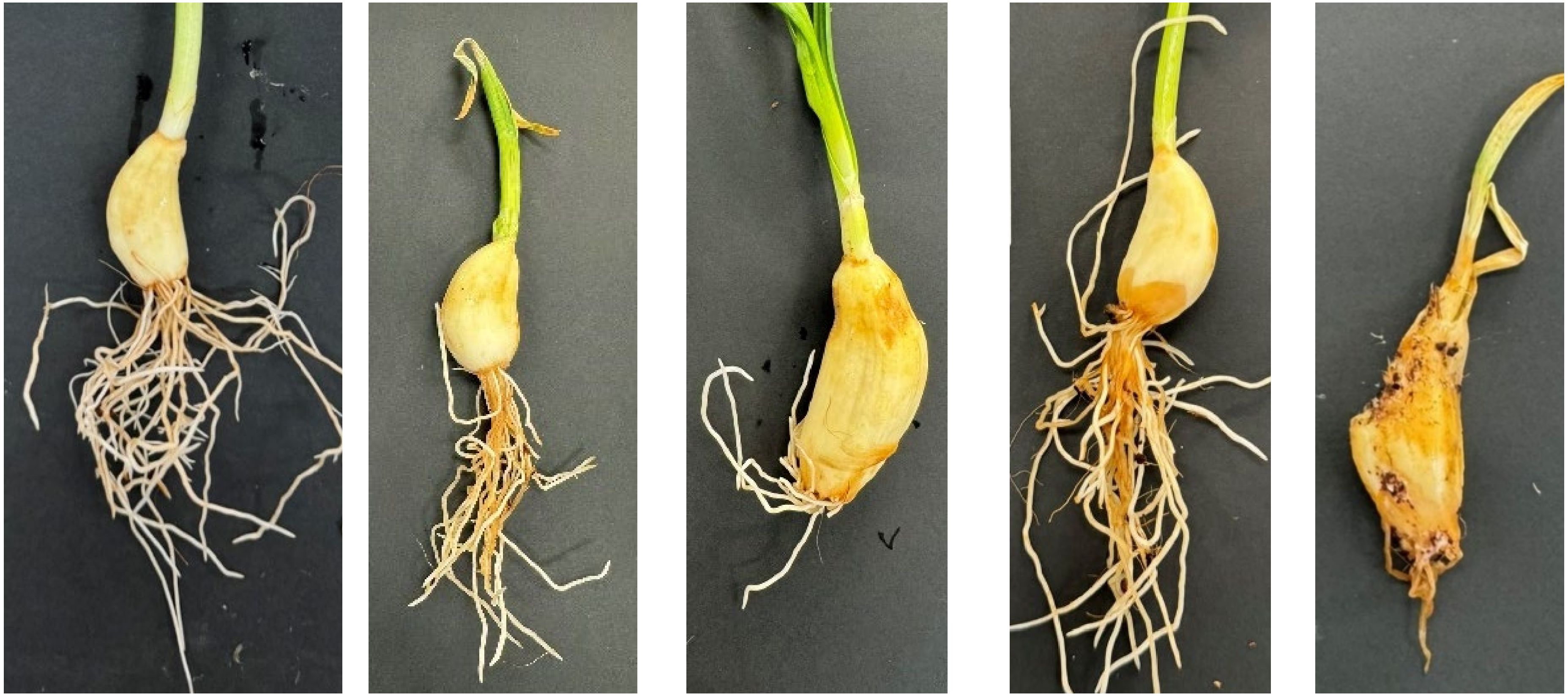

(a) (b) (c) (d) (e)

Figure S2. Experiment 3 disease rating results of FP and FO *ex* garlic in garlic seedlings. Disease severity was assessed using a rating scale from 0 to 4. **(a)** DR 0 – No disease symptoms. **(b)** DR 1 – Mild symptoms included up to 10% of the clove having discolouration and usually at the baseplate. **(c)** DR 2 – Moderate symptoms consisted of ~10-30% of clove having rot symptoms. **(d)** DR 3 – Moderate to severe symptoms consisted of cloves with 30-50% of the clove having discolouration/rotting symptoms. **(e)** DR 4 – Highly severe disease symptoms consisted of 50-100% rotting of the clove.

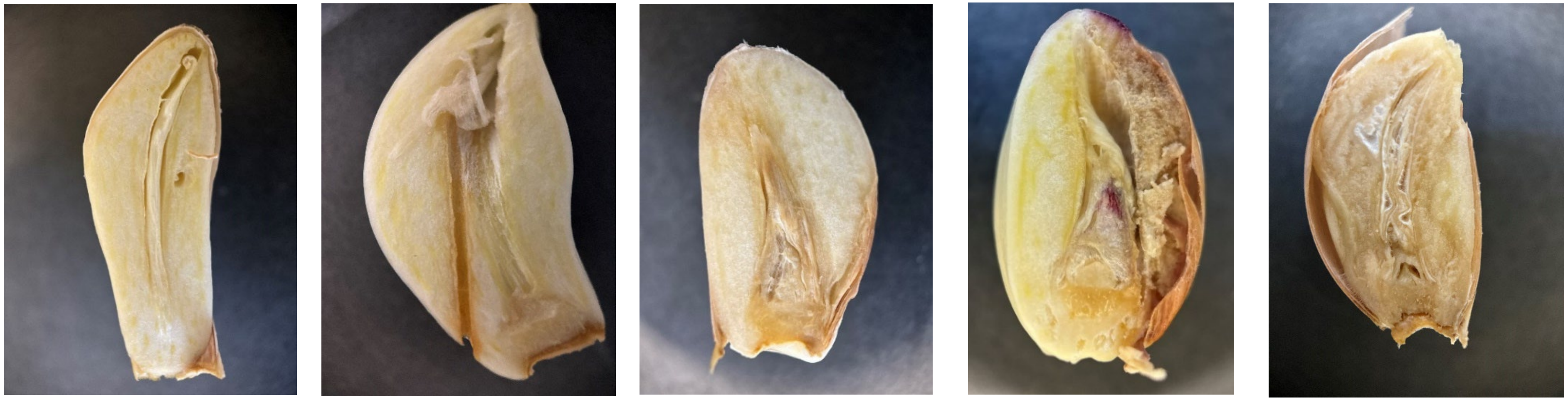

(a) (b) (c) (d) (e)

Figure S3. Disease ratings for Experiment 4 results for pathogenicity tests of FO *ex* garlic and FOC *ex* onion on garlic cloves. Disease severity was assessed using a rating scale from 0 to 4. **(a)** DR 0 – No disease symptoms. **(b)** DR 1 – Mild symptoms included discolouration at the site of injection (up to 10% of the clove displaying discolouration). **(c)** DR 2 – Moderate symptoms consisted of discolouration beyond the site of injection (~10-30% of clove having rot symptoms). **(d)** DR 3 – Moderate to severe symptoms consisted of cloves with 30-50% of the clove having discolouration/rotting symptoms and severe symptoms. **(e)** DR 4 – Highly severe disease symptoms consisted of 50-100% rotting of the clove.

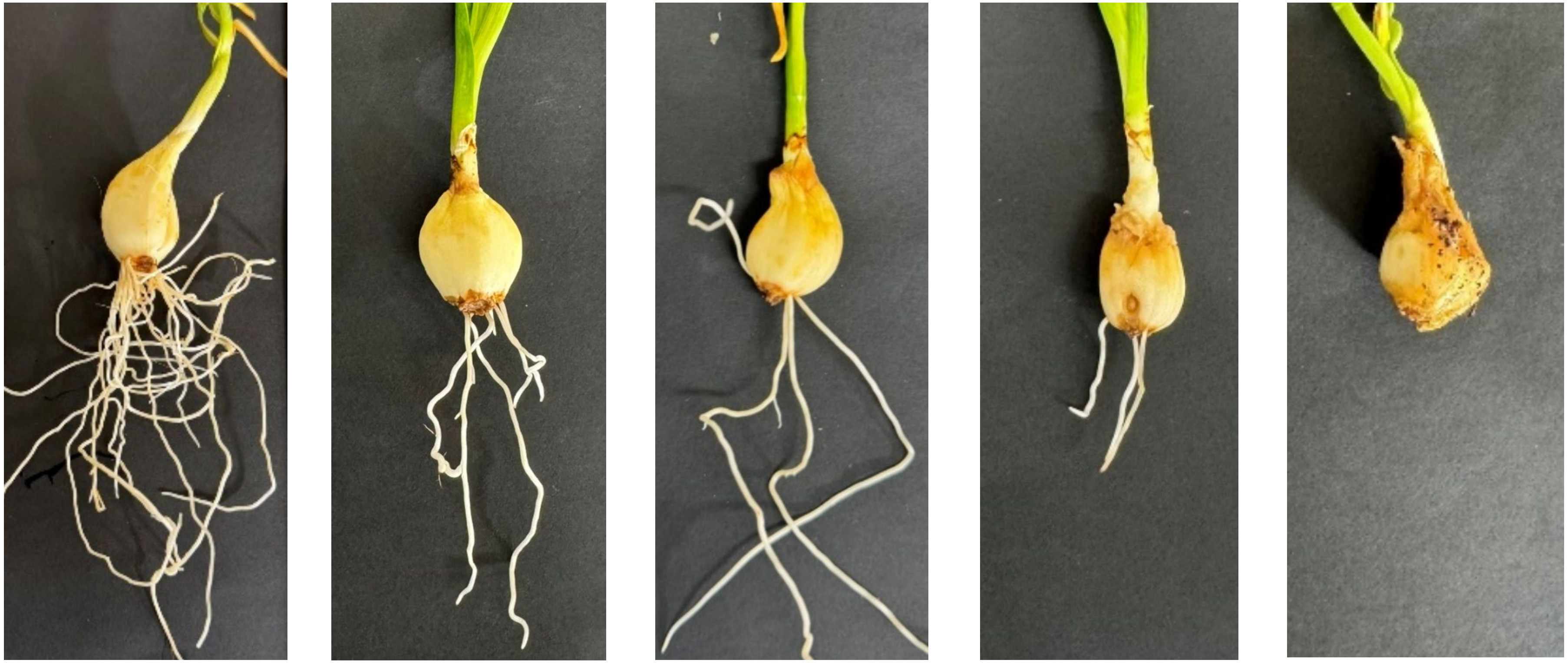

(a) (b) (c) (d) (e)

Figure S4. Disease ratings for Experiment 5 results for pathogenicity tests of FO *ex* garlic and FOC *ex* onion on garlic seedlings. Disease severity was assessed using a rating scale from 0 to 4. **(a)** DR 0 – No disease symptoms. **(b)** DR 1 – Mild symptoms included up to 10% of the clove having discolouration and usually at the baseplate. **(c)** DR 2 – Moderate symptoms consisted of ~10-30% of clove having rot symptoms. **(d)** DR 3 – Moderate to severe symptoms consisted of cloves with 30-50% of the clove having discolouration/rotting symptoms. **(e)** DR 4 – Highly severe disease symptoms consisted of 50-100% rotting of the clove.

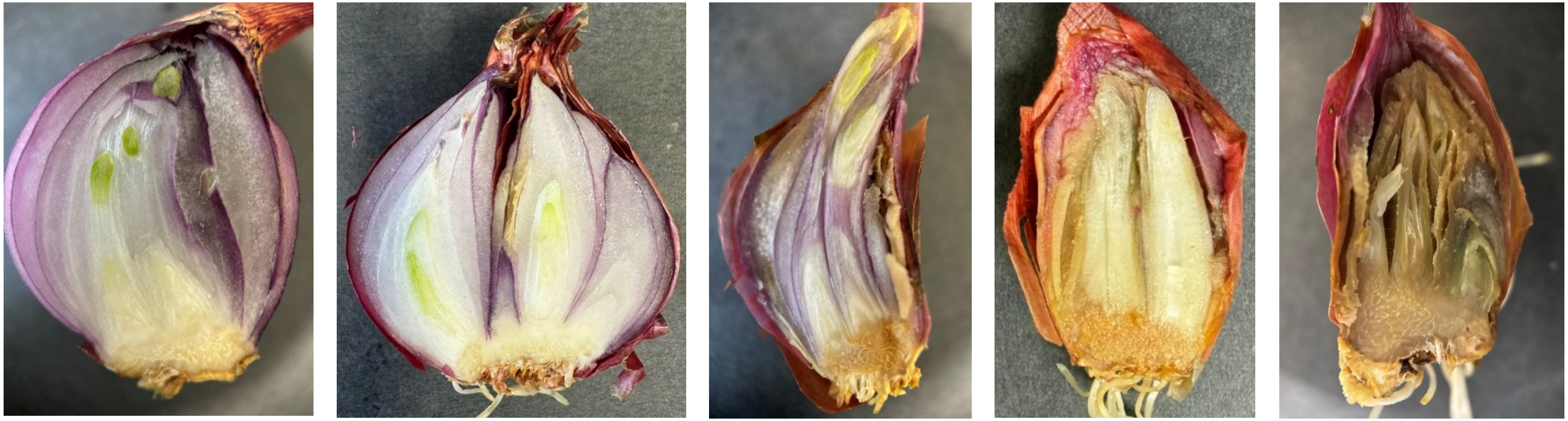

(a) (b) (c) (d) (e)

Figure S5. Experiment 6 results for disease ratings for pathogenicity tests of FO *ex* garlic and FOC *ex* onion on mature shallot bulbs. Disease severity was assessed using a rating scale from 0 to 4. **(a)** DR 0 – No disease symptoms. **(b)** DR 1 – Mild symptoms included discolouration at the site of injection (up to 10% of the bulb displaying discolouration). **(c)** DR 2 – Moderate symptoms consisted of discolouration beyond the site of injection (~10-30% of bulb having rot symptoms). **(d)** DR 3 – Moderate to severe symptoms consisted of bulbs with 30-50% of the clove having discolouration/rotting symptoms. **(e)** DR 4 – Highly severe disease symptoms consisted of 50%-100% rotting of the bulb.

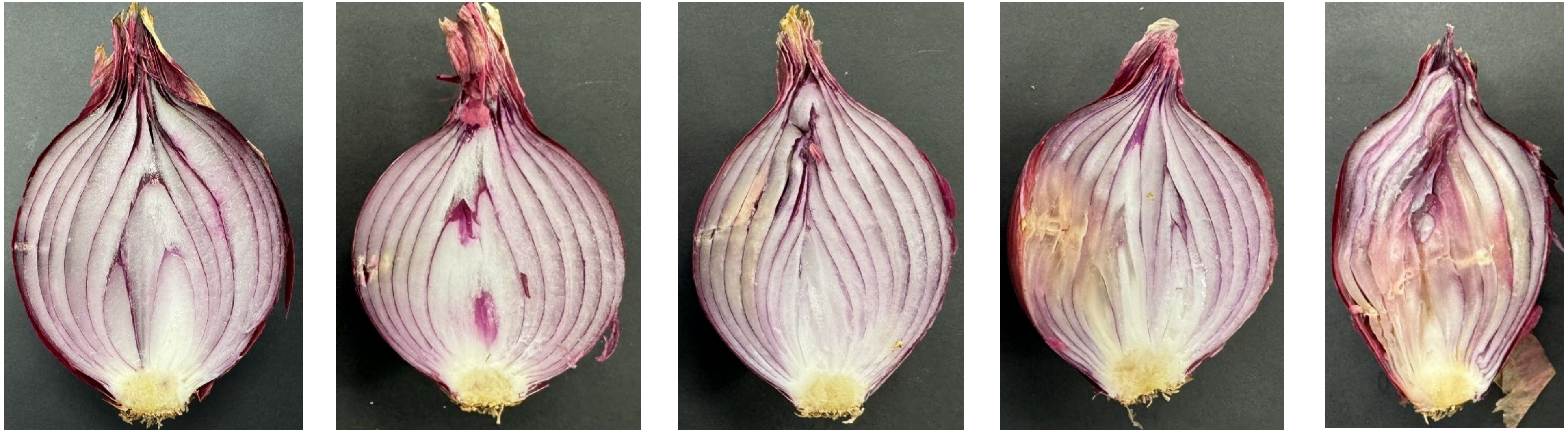

(a) (b) (c) (d) (e)

Figure S6. Experiment 7 results for disease ratings for pathogenicity tests of FO *ex* garlic and FOC *ex* onion on mature onion bulbs. Disease severity was assessed using a rating scale from 0 to 4. **(a)** DR 0 – No disease symptoms. **(b)** DR 1 – Mild symptoms included discolouration at the site of injection (up to 10% of the bulb displaying discolouration). **(c)** DR 2 – Moderate symptoms consisted of discolouration beyond the site of injection (~10-30% of bulb having rot symptoms). **(d)** DR 3 – Moderate to severe symptoms consisted of bulbs with 30-50% of the clove having discolouration/rotting symptoms. **(e)** DR 4 – Highly severe disease symptoms consisted of 50%-100% rotting of the bulb.

Table S2. Reference sequences for BLASTn search of *Fusarium* genomes included in this study.

Gene	NCBI accession	Accession details	Reference
TEF	AF008489.1	*Fusarium oxysporum* f. sp. cubense NRRL 25607	[26]
RPB1	KC808302.1	*Fusarium oxysporum* culture NRRL:62542	[61]
RPB2	KP964845.1	*Fusarium oxysporum* f. sp. *cepae* isolate HAZ	[12]
TUB	KP964910.1	*Fusarium oxysporum* isolate A13	[12]
SIX1	GQ268948.1	*Fusarium oxysporum* f. sp. *lycopersici* isolate BFOL-51	[19]
SIX2-1	OL763387.1	*Fusarium proliferatum* isolate Fpr047	[15]
SIX2-2	OL763389.1	*Fusarium proliferatum* isolate Fpr057	[15]
SIX3	GQ268955.1	*Fusarium oxysporum* f. sp. *lycopersici* isolate 14844	[19]
SIX4	GQ268951.1	*Fusarium oxysporum* f. sp. *lycopersici* isolate BFOL-51	[19]
SIX5	KP964966.1	*Fusarium oxysporum* f. sp. *cepae* isolate A21	[12]
SIX6	GQ268953.1	*Fusarium oxysporum* f. sp. *lycopersici* isolate BFOL-51	[19]
SIX7	GQ268954.1	*Fusarium oxysporum* f. sp. *lycopersici* isolate BFOL-51	[19]
SIX8	FJ755837.1	*Fusarium oxysporum* f. sp. *lycopersici*	Rep, M. 2009 Unpublished
SIX9	KX435017.1	*Fusarium oxysporum* f. sp. *cubense* strain NRRL25609	[49]
SIX10	MW939421.1	*Fusarium oxysporum* f. sp. *cepae* isolate Fox129	[15]
SIX11	KX435049.1	*Fusarium oxysporum* f. sp. *passiflorae* strain BRIP28044	[49]
SIX12	MT710734.1	*Fusarium oxysporum* f. sp. *pisi* isolate FOP1	[62]
SIX13	KX435044.1	*Fusarium oxysporum* f. sp. *niveum*	[49]
SIX14	MK906696.1	*Fusarium oxysporum* isolate Fol1	Batson, A.M. and du Toit, L.J. 2019 Unpublished
C5	MW939410.1	*Fusarium oxysporum* f. sp. *cepae* isolate Fox220a	[14]
CRX1	KP965011.1	*Fusarium oxysporum* f. sp. *cepae* isolate FUS2	[12]
CRX2Fp	OP490290.1	*Fusarium proliferatum* isolate FUS15343	[15]
CRX2Fo	OP490286.1	*Fusarium oxysporum* f. sp. *cepae* isolate FUS15317	[15]
FUM1	KF415130.1	*Fusarium proliferatum* strain ITEM 2287	[63]

Table S3. *Fusarium* spp. genomes downloaded from GenBank and used in phylogenetic analysis.

Name	GenBank Accession
F. avenaceum_WV21P1A	CP109663.1-CP109671.1
F. verticillioides_7600	AAIM00000000
FO_albedinis_Foa 44	JACSDM010000000
FO_apii_207.A	JAAOOO010000000
FO_apii_NRRL38295	JAAOOP000000000
FO_basilici_VPRI44259	JAMSFS010000000
FO_basilici_VPRI44260	JAMSFR010000000
FO_cepae_FoC_125	MRCV01000000
FO_cepae_FoC_A23	MRCW01000000
FO_cepae_FoC_Fus2	MRCU01000000
FO_ciceris_38-1	MEHF01000000
FO_conglutinans_R2_54008	AGNF01000000
FO_conglutinans_VPRI44253	JAMSGH000000000
FO_coriandrii_GL306	JAAOOM010000000
FO_cubense_C1HIR_9889	MBFV00000000
FO_cubense_Foc-UH	JAUEPI010000000
FO_cubense_R1_TC1-1	VLOF03000000
FO_cubense_R1_VCG01220	JAEMVU010000000
FO_cubense_TR4	JAJNCA000000000
FO_cubense_VCG0125	JAEMVW010000000
FO_cucumerinum_Foc001	MAKZ01000000
FO_cucumerinum_Foc015	MABK01000000
FO_Fo47	CP052038-CP052049.1
FO_Fo5176	CP128282-CP128299.1
FO_fragariae_GL1381	WIMJ00000000
FO_fragariae_MAFF727510	WILW02000000
FO_fragariae_N-17203	WILP01000000
FO_fragariae_Nasushiobara1	WILM01000000
FO_gladioli_G2	NJCL01000000
FO_gladioli_G76	NJCK01000000
FO_koae_44	CP052897-CP052908.1
FO_lagenariae_03-05118	NJCI01000000
FO_lagenariae_Lag:1-1	NJCG00000000
FO_lilii_Fol39	NJCF01000000
FO_lini_39	JABJUA010000000
FO_lini_F287	JABJUC010000000
FO_luffae_Fol-167	NJCD01000000
FO_lycopersici_4287	QESU01000000
FO_lycopersici_4287	AAXH00000000
FO_lycopersici_Fol007	MALI00000000

FO_lycopersici_MN25	AGBH01000000
FO_lycopersici_R3_D11	RBXW00000000
FO_lycopersici_VPRI44100	JAMSEP010000000
FO_lycopersici_VPRI44272	JAMSFG010000000
FO_matthiolae_Stocks4	JAJGYP010000000
FO_medicaginis_VPRI44256	JAMSFV010000000
FO_melonis_26406	AGNE00000000
FO_melonis_Fom004	MALX01000000
FO_melonis_NRRL 26172	JAAIIO010000000
FO_momordicae_NRRL26413	NJCB01000000
FO_narcissi_Na5	NJCV01000000
FO_nicotianae_Ft-Rob	NJBX01000000
FO_niveum_Fon005	MAKY01000000
FO_niveum_Fon013	MALC01000000
FO_niveum_VPRI44306	JAMSDS000000000
FO_passiflorae_VPRI44203	JAMSGA010000000
FO_passiflorae_VPRI44302	JAMSDW010000000
FO_physali_B01	JADWPC010000000
FO_physali_B117	JADWOZ010000000
FO_pisi_HDV247	AGBI01000000
FO_radicis-cucumerinum_Forc024	MABR01000000
FO_raphani_54005	AGNG01000000
FO_raphani_Tf1262	JAELUR010000000
FO_spinaciae_Fus167	JAALGP020000000
FO_spinaciae_Fus254	JAALGI000000000
FO_spinaciae_MF15	JABCQZ010000000
FO_tracheiphilum_VPRI44258	JAMSFT010000000
FO_tulipae_VPRI44264	JAMSFN000000000
FO_tulipae_VPRI44266	JAMSFL000000000
FO_vasinfectum_25433	AGNC00000000
FO_vasinfectum_ME23	CP130297-CP130309.1
FO_vasinfectum_NRRL 25432	JAANYL010000000
FO_vasinfectum_TF1	VINL01000000
FO_vasinfectum_VPRI44283	JAMSDL000000000
FO_zingiberi_VPRI44200	JAMSFW010000000
FO_zingiberi_VPRI44278	JAMSFA010000000
FP_Fp_A8	MRDB01000000
FP_KF3377	PKMH01000000
FP_MPVP 328	PKMJ01000000

FP_R16	PKMG01000000

Table S4. *SIX2*, *SIX9*, *SIX13*, *CRX1*, *CRX2*, *C5*, and *FUM1* sequence accessions downloaded from GenBank and used in phylogenetic analysis.

Name	Gene	Accession
FO_lycopersici_Fol59_*SIX2*	*SIX2*	MN745205.1

FO_lycopersici_Fol_UDC-10_*SIX2*	*SIX2*	MN745206.1
FP_FUS16163 _*SIX2*-1	*SIX2*	OL763385.1
FP_Fpr057_*SIX2*-1	*SIX2*	OL763386.1
FP_FUS16059_*SIX2*-1	*SIX2*	OL763383.1
FP_FUS16091_*SIX2*-1	*SIX2*	OL763384.1
FP_Fpr047_*SIX2*-1	*SIX2*	OL763387.1
FO_cubense_BRIP44012_*SIX2*	*SIX2*	KX435003.1
FO_cubense_BRIP40340_*SIX2*	*SIX2*	KX435000.1
FP_FUS16163_*SIX2*-2	*SIX2*	OL763388.1
FP_Fpr057_*SIX2*-2	*SIX2*	OL763389.1
FO_UQ6516_*SIX2*	*SIX2*	MW076669.1
F. sacchari_UQ6549_*SIX2*	*SIX2*	MW076672.1
F. sacchari_UQ6569_*SIX2*	*SIX2*	MW076666.1
F. sacchari_UQ6587_*SIX2*	*SIX2*	MW076668.1
F. sacchari _UQ66174_*SIX2*	*SIX2*	MW076675.1
FO_UQ6581_*SIX2*	*SIX2*	MW076688.1
FO_UQ6580_*SIX2*	*SIX2*	MW076681.1
FO_niveum_RBG5771_*SIX9*	*SIX9*	KR855725.1
FO_niveum_FRL1583_*SIX9*	*SIX9*	KR855728.1
FO_niveum_FRL4120_*SIX9*	*SIX9*	KR855729.1
FO_lycopersici_Fol007_*SIX9*	*SIX9*	KC701447.1
FO_lycopersici_FRL4273_*SIX9*	*SIX9*	KR855730.1
FO_lycopersici_RBG5768_*SIX9*	*SIX9*	KR855731.1
F. commune_R-1_*SIX9*	*SIX9*	LC739705.1
FO_passiflorae_FRL1584_*SIX9*	*SIX9*	KR855722.1
FO_passiflorae_RBG5775_*SIX9*	*SIX9*	KR855723.1
FO_passiflorae_RBG6380_*SIX9*	*SIX9*	KR855724.1
FO_passiflorae_BRIP28044_*SIX9*	*SIX9*	KX435047.1
FO_passiflorae_06603F_*SIX9*	*SIX9*	MW328516.1
FO_cubense_NRRL25609_*SIX9*	*SIX9*	KX435017.1
FO_cubense_BRIP63259_*SIX9*	*SIX9*	KX435016.1
FO_cepae_Fox260_*SIX9*	*SIX9*	MW939406.1
FO_cepae_Fox220a_*SIX9*	*SIX9*	MW939414.1
FO_cepae_Fox129_*SIX9*	*SIX9*	MW939420.1
FO_Fus129_*SIX9*	*SIX9*	MK906664.1
FO_fragariae_BRIP62122a_*SIX13*	*SIX13*	OR743728.1
F. sacchari__UQ6616_*SIX13*	*SIX13*	MW076820.1
FO_fragariae_BRIP53860_*SIX13*	*SIX13*	KX435029.1
FO_lini_FOLIN_*SIX13*	*SIX13*	KP964998.1
FO_physali_MAP5_*SIX13*	*SIX13*	MT738929.1
FO_lycopersici_Fol007_*SIX13*	*SIX13*	KC701451.1
FO_niveum_NTDPI36955_*SIX13*	*SIX13*	KX435043.1
FO_niveum_NTDPI36955_*SIX13*	*SIX13*	KX435044.1
FO_cepae_FUS15317_*CRX1*	*CRX1*	OP490283.1

FO_FUS15367_*CRX1*	*CRX1*	OP490284.1
FP_FUS15343_*CRX2*	*CRX2*	OP490290.1
FP_FUS16091_*CRX2*	*CRX2*	OP490293.1
FP_FUS16171_*CRX2*	*CRX2*	OP490294.1
FP_FUS15329_*CRX2*	*CRX2*	OP490289.1
FP_FUS16059_*CRX2*	*CRX2*	OP490292.1
FP_FUS15373_*CRX2*	*CRX2*	OP490291.1
FO_cepae_Fox220a_*C5*	*C5*	MW939410.1
FO_cepae_Fox260_*C5*	*C5*	MW939402.1
F. verticillioides_NOS2_*FUM1*	*FUM1*	DQ384219.1
FO_O-1890_*FUM1*-like	*FUM1*	AY577457.1
F. anthophilum_NRRL 25214_*FUM1*-like	*FUM1*	AY577453.1
F. globosum_NRRL 26133_*FUM1*-like	*FUM1*	AY577455.1
F. fujikuroi_M-6884_*FUM1*-like	*FUM1*	AY577454.1
F. fujikuroi_HKM 41 _*FUM1*	*FUM1*	KF415129.1
FP_Fpro-277, clone OM6_*FUM1*	*FUM1*	AM945866.1
FP_Fpro-271, clone OM5_*FUM1*	*FUM1*	AM945865.1
FP_Fpro-227, clone OM24_*FUM1*	*FUM1*	AM945859.1
FP_Fpro-279, clone OM7_*FUM1*	*FUM1*	AM945867.1
FP_Fpro-241, clone OM25_*FUM1*	*FUM1*	AM945862.1
FP_Fpro-227, clone 4-2_*FUM1*	*FUM1*	AM945858.1
FP_ITEM 2287 _*FUM1*	*FUM1*	KF415130.1
FP_Fpro-230, clone OM1_*FUM1*	*FUM1*	AM945860.1
FP_M-6993_*FUM1*	*FUM1*	AY577458.1
